# Conversion from Tacrolimus to Cyclosporine A Improves Glucose Tolerance in HCV-Positive Renal Transplant Recipients

**DOI:** 10.1371/journal.pone.0145319

**Published:** 2016-01-06

**Authors:** Ammon Handisurya, Corinna Kerscher, Andrea Tura, Harald Herkner, Berit Anna Payer, Mattias Mandorfer, Johannes Werzowa, Wolfgang Winnicki, Thomas Reiberger, Alexandra Kautzky-Willer, Giovanni Pacini, Marcus Säemann, Alice Schmidt

**Affiliations:** 1 Department of Internal Medicine III, Division of Nephrology and Dialysis, Medical University of Vienna, Vienna, Austria; 2 Institute of Neurosciences, CNR, Padova, Italy; 3 Department of Emergency Medicine, Medical University of Vienna, Vienna, Austria; 4 Department of Internal Medicine III, Division of Gastroenterology and Hepatology, Medical University of Vienna, Vienna, Austria; 5 Department of Internal Medicine III, Division of Endocrinology and Metabolism, Medical University of Vienna, Vienna, Austria; Clínica Universidad de Navarra, SPAIN

## Abstract

**Background:**

Calcineurin-inhibitors and hepatitis C virus (HCV) infection increase the risk of post-transplant diabetes mellitus. Chronic HCV infection promotes insulin resistance rather than beta-cell dysfunction. The objective was to elucidate whether a conversion from tacrolimus to cyclosporine A affects fasting and/or dynamic insulin sensitivity, insulin secretion or all in HCV-positive renal transplant recipients.

**Methods:**

In this prospective, single-center study 10 HCV-positive renal transplant recipients underwent 2h-75g-oral glucose tolerance tests before and three months after the conversion of immunosuppression from tacrolimus to cyclosporine A. Established oral glucose tolerance test-based parameters of fasting and dynamic insulin sensitivity and insulin secretion were calculated. Data are expressed as median (IQR).

**Results:**

After conversion, both fasting and challenged glucose levels decreased significantly. This was mainly attributable to a significant amelioration of post-prandial dynamic glucose sensitivity as measured by the oral glucose sensitivity-index OGIS [422.17 (370.82–441.92) vs. 468.80 (414.27–488.57) mL/min/m2, p = 0.005), which also resulted in significant improvements of the disposition index (p = 0.017) and adaptation index (p = 0.017) as markers of overall glucose tolerance and beta-cell function. Fasting insulin sensitivity (p = 0.721), insulinogenic index as marker of first-phase insulin secretion [0.064 (0.032–0.106) vs. 0.083 (0.054–0.144) nmol/mmol, p = 0.093) and hepatic insulin extraction (p = 0.646) remained unaltered. No changes of plasma HCV-RNA levels (p = 0.285) or liver stiffness (hepatic fibrosis and necroinflammation, p = 0.463) were observed after the conversion of immunosuppression.

**Conclusions:**

HCV-positive renal transplant recipients show significantly improved glucose-stimulated insulin sensitivity and overall glucose tolerance after conversion from tacrolimus to cyclosporine A. Considering the HCV-induced insulin resistance, HCV-positive renal transplant recipients may benefit from a cyclosporine A-based immunosuppressive regimen.

**Trial Registration:**

ClinicalTrials.gov NCT02108301

## Introduction

Post-transplant diabetes mellitus (PTDM) affects 5–35% of all renal transplant recipients (RTRs) and leads to an attenuated graft function, reduced graft and patient survival and increased cardiovascular mortality [1–4]. Hepatitis C-virus (HCV) infection and the use of immunosuppressants, especially the calcineurin-inhibitors (CNIs) tacrolimus (TAC) and cyclosporine A (CyA), strongly increase the risk to develop PTDM [1–3].

Although an increased diabetogenicity of TAC compared to CyA is generally acknowledged, the detailed pathophysiological mechanisms underlying the distinct glucometabolic effects of both CNIs, i.e. the differential regulation of insulin sensitivity and/or insulin secretion by TAC and CyA, remain to be determined. The vast majority of previous studies evaluating glucose tolerance in RTRs employed parameters of fasting insulin sensitivity, like the homeostasis model assessment of insulin resistance (HOMA-IR) or the quantitative insulin sensitivity check-index (QUICKI), but not of dynamic/glucose-stimulated insulin sensitivity and insulin secretion. This, however, is pivotal for the evaluation of glucose tolerance as fasting insulin sensitivity primarily reflects hepatic insulin resistance but not insulin resistance of two other major insulin-sensitive tissues skeletal muscle and adipose tissue [5], which warrant parameters of glucose-stimulated insulin sensitivity, like the M/I-value derived from euglycemic-hyperinsulinemic clamps or e.g. the oral glucose sensitivity (OGIS) index derived from intravenous or oral glucose tolerance tests (OGTT).

Several studies provided strong evidence that chronic HCV infection promotes insulin resistance directly via interference with the intracellular insulin signalling cascade, increase of proinflammatory cytokines or downregulation of the glucose transporter-4 and -2 in skeletal muscle and liver. In addition, HCV induces hepatic fibrosis, which itself facilitates insulin resistance [6]. Of note, chronic HCV infection induces insulin resistance not only in the liver but predominantly in skeletal muscle [7], which is not adequately assessed by parameters of fasting insulin sensitivity like HOMA-IR or QUICKI. HCV-positive patients including RTRs present a high prevalence of insulin resistance with, in part, compensatorily increased insulin secretion [6–9]. Interestingly, insulin resistance improves in subjects following eradication of HCV, but not in virological non-responders [10]. With regard to HCV-treatment, CyA but not TAC was recently found to decrease HCV-RNA and HCV protein production at levels >1000 ng/mL *in vitro* [11] and, when administered in combination with interferon-alpha, to result in higher rates of sustained virological response than interferon alpha alone [12].

The objective of this study was to prospectively evaluate whether the conversion from TAC to CyA (i) alters glucose metabolism in HCV-positive RTRs and (ii) to assess potential underlying mechanisms of fasting and dynamic insulin sensitivity and insulin secretion.

## Materials and Methods

### Study participants

**Fig 1:**
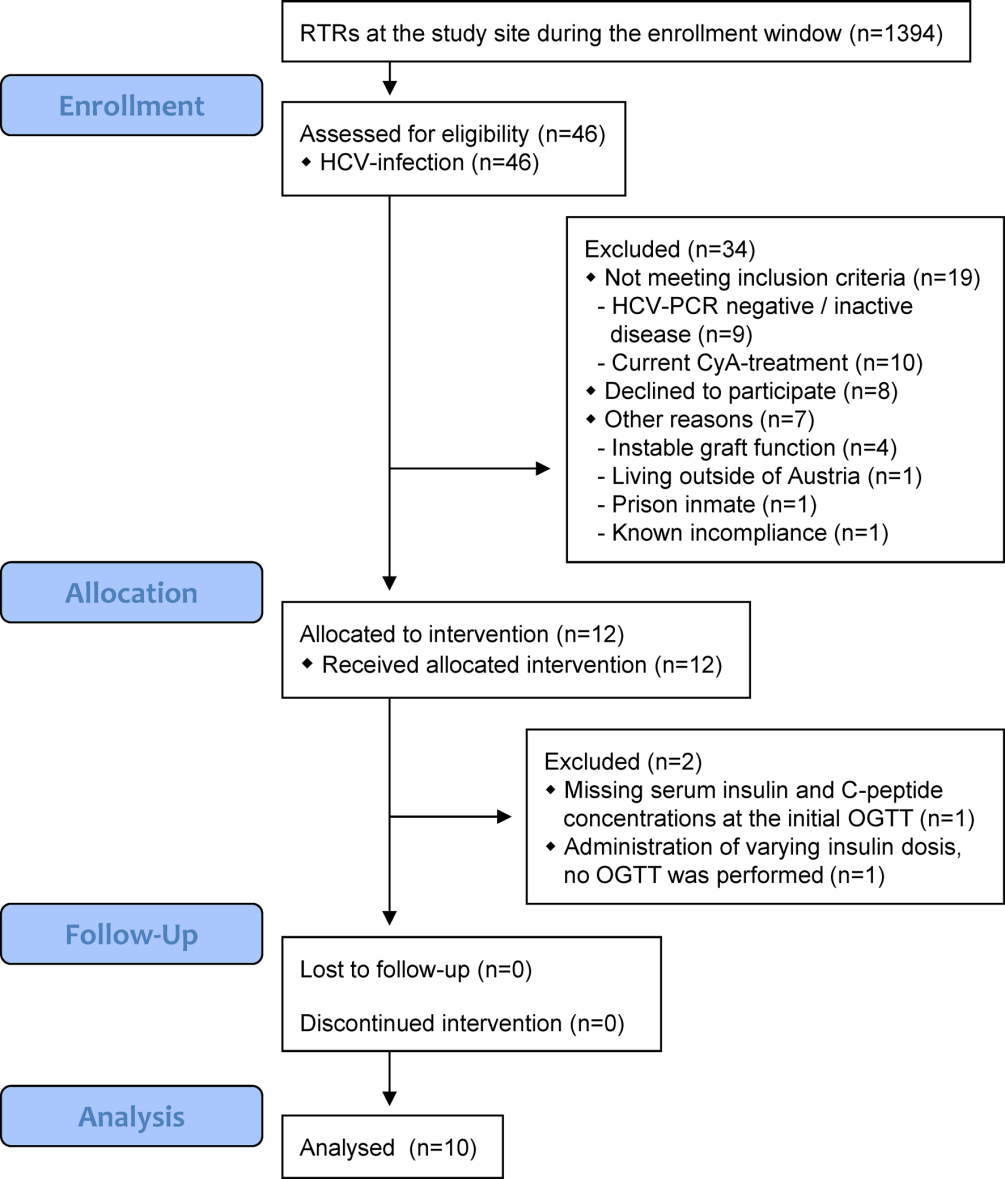
Trend flow chart.

In this prospective, single-center, open study, all HCV-positive RTRs (n = 46, including 13 subjects with known PTDM, two with pre-transplant type 2 diabetes mellitus and 31 without known overt diabetes mellitus), who were admitted at the outpatient department of the Division of Nephrology and Dialysis, Department of Medicine III, Medical University of Vienna, between 01-July-2011 and 31-Aug 2012, were assessed for eligibility (Fig 1). Inclusion criteria comprised written informed consent, prior renal transplantation, current treatment with TAC, HCV infection and age 18–70 years. Subjects with known CyA-intolerance or current renal replacement therapy and pregnant or breastfeeding women were excluded. Twelve patients fulfilled all inclusion and exclusion criteria. One subject was excluded two days after initiation of CyA-treatment due to missing insulin and C-peptide concentrations at the initial OGTT and one subject was excluded due to varying subcutaneous insulin doses (conversion of immunosuppression, but no OGTT was performed). Ten HCV-positive RTRs (sex: 8 male, 2 female) completed the study (Fig 1); three of which had known PTDM (one treated with the DPP4-inhibitor sitagliptin, one with pre-mixed insulin, one dietary alone; see below). The distribution of HCV-genotypes was: 2 genotype 1a, 5 genotype 1b, 1 genotype 1b/3a, 1 genotype 4, 1 genotype 4a/4c/4d. No subject received any specific anti-HCV treatment since transplantation including the study period itself. The modality of dialysis prior to transplantation was: 1 pre-emptive, 7 hemodialysis only, 1 peritoneal dialysis only, 2 peritoneal dialysis followed by hemodialysis. Four of nine study participants (one missing data) were pre-sensitized [latest complement-dependent cytotoxicity–panel reactive antibodies (CDC-PRA) >10%]. Renal graft function was stable in all subjects in the six months prior to study inclusion and all study participants showed no signs of infection or other conditions (such as malignancy or cardiovascular events), which may influence glucose homeostasis.

### Study design

After an 12 hours’ overnight fast, 2h-75g-OGTTs with measurement of glucose, insulin and C-peptide levels at 0/30/60/90/120 minutes were performed in 10 HCV-positive RTRs before and three months after the conversion of immunosuppression from daily oral TAC [Prograf capsules (twice daily) or Advagraf capsules (once daily), Astellas Pharma GmBH] to twice daily oral CyA (Sandimmun capsules, Novartis GmBH). In an effort to decrease plasma HCV-RNA levels, CyA was initially titrated to the highest tolerated dose aiming at C2-levels of 416.4–833.0 nmoL/L and starting with 3–5 mg/kg/d (limited by an increase of serum creatinine and bilirubin by more than 20% from the pre-conversion level); three and ten days after the conversion of immunosuppression median CyA-trough levels were 89.64 (83.30–125.78) nmol/L and 167.85 (117.04–203.67) nmol/L, respectively, and median C2-levels were 506.46 (458.98–534.99) nmol/L and 619.34 (550.82–762.20) nmol/L, respectively. To avoid possible influences on glucose metabolism, concomitant immunosuppressants [mycophenolate acid (mean dose: 762.0±175.2 mg/d, range: 0–1500 mg/d), steroids (mean prednisone dose: 2.88±0.75 mg/d; range: 0–5 mg/d] and non-immunosuppressive medication known to influence glucose tolerance including DPP4-inhibitors, pre-mixed insulin, beta-blocking substances, renin-angiotensin-inhibitors, diuretics, statins, antidepressants/neuroleptics and levothyroxine-sodium, remained unaltered during the study period. Due to the potentially differential inter-individual resorption and the known alteration of glucose tolerance by CNIs the morning dose of the immunosuppressants was withheld until after the completion of the OGTT (trough level-conditions). Similarily, the morning dose of sitapliptin and pre-mixed insulin was withheld in the two subjects with known PTDM (last administration >24 hours and >12 hours, respectively).

Plasma concentrations of TAC and CyA were measured using CMIA and ACMIA, respectively, at certified laboratories of the Department of Laboratory Medicine, Medical University of Vienna.

#### Estimation of basal and dynamic insulin sensitivity and insulin secretion

Established OGTT-derived, model-based indices were employed to evaluate glucose tolerance. From glucose, insulin and C-peptide concentration measurements the following parameters of insulin kinetics were obtained: area under the curve (AUC) for glucose, insulin and C-peptide (by trapezoidal integration), total beta-cell insulin secretion rate (TIS), its suprabasal component (dynamicTIS) and hepatic insulin extraction (HIE) [13]. Dynamic insulin sensitivity was estimated with OGIS, which describes the glucose clearance per unit change of insulin concentration [14,15], and fasting insulin sensitivity with QUICKI. Beta-cell function was evaluated with IGI, calculated as the ratio of 30 to 0 min insulin difference to the same difference in glucose, i.e. 30–0 delta insulin / 30–0 delta glucose [16]. Beta-cell compensatory mechanisms for changes of insulin resistance were quantified by the products DI = OGIS×IGI (called disposition index) and AI = OGIS×IGI_C-peptide_ (termed adaptation index) [14,15,17,18], both representing additional markers of overall glucose metabolism. Based on the measurement of C-peptide, which is secreted equimolarily with insulin but which does not underlie a hepatic first-pass effect, AI yields an estimation of the pre-hepatic insulin-secretory capacity of pancreatic beta-cells, i.e. unaffected by HIE, in response to the prevailing insulin sensitivity [14], whereas the insulin-based DI relates the post-hepatic insulin response to changes in insulin sensitivity [14]. All parameters were validated in the non-transplant population and OGIS also recently in RTRs [19]. OGTTs are considered gold-standard for the diagnosis of PTDM [19].

#### Estimation of renal and hepatic function

Estimated glomerular filtration rate (eGFR) was calculated according to the modification of diet in renal disease (MDRD)-formula.

Hepatic necroinflammatoin and function was estimated by measurement of standard biochemical liver parameters. For better characterization of our study collective, the Fib-4 index, a non-invasive score to assess liver fibrosis with was calculated as [age (years) x AST (U/L)] / [platelets (10^9^/L) x ALT (U/L)^½^] [20].

In addition, liver stiffness was assessed by transient elastography (FibroScan, EchoSens, Paris, France) in eight subjects, as described previously [21]. Briefly, an ultrasonic transducer transmits a vibration wave with low frequency (50 MHz) and amplitude through the right liver lobe with the patient lying in the dorsal decubitus position with the right arm in maximal abduction. The velocity of this vibration wave correlates directly with the hepatic stiffness. Transient elastography is a thus a rapid and non-invasive method to assess the degree of liver fibrosis and is well-validated for HCV-positive subjects.

### Approval and Registration

The study protocol was performed in accordance to the declaration of Helsinki and approved by the Ethics Committee of the Medical University of Vienna (EK-No. 477/2011) and by the Austrian Federal Office for Safety in Health Care (AGES). The clinical and research activities being reported are consistent with the Principles of the Declaration of Istanbul as outlined in the “Declaration of Istanbul on Organ Trafficking and Transplant Tourism”. All study participants gave written informed consent. The study was registered at ClinicalTrials.gov (no. NCT02108301) on 01-Apr-2014.

### Statistical analysis

The primary outcome variable was dynamic insulin sensitivity parameter OGIS; secondary outcome parameters included QUICKI, IGI, DI and AI. Wilcoxon signed-rank test was used to evaluate the intra-individual effects of the CNI-conversion on primary and secondary outcome variables as well as supportive parameters, including parameters of renal and hepatic function, before as compared to after conversion of immunosuppression. Spearman’s correlation coefficients were applied. Mann-Whitney U Test was used to assess differences in changes (Δ) of the primary and secondary outcome parameters between subjects with PTDM and those with normal glucose tolerance. Statistical analysis was performed using SAS Enterprise Guide 4.2 (SAS Institute Inc., Cary, NC) and IBM SPSS v23 statistical software (IBM Corp., Armonk, NY). Data are expressed as median (IQR). p<0.05 was considered statistically significant.

Considering insulin sensitivity as the primary endpoint, a post-hoc analysis found that a sample size of 10 had 99% power to detect a difference in OGIS of 47 ml min^-1^m^-2^, with a standard deviation of 31 using a paired *t*-test with a 0.05 two-sided significance level.

## Results

### Baseline characteristics

Compared to baseline values under the initial TAC-treatment, no significant differences in BMI, eGFR, urinary protein-creatinine-ratio, serum concentrations of creatinine, albumin, HDL-C, LDL-C, C-reactive protein, or thyroid hormones were observed three months after the conversion to CyA (Table 1). Serum triglyceride and total cholesterol levels increased, while alanine aminotransferase and aspartate aminotransferase decreased significantly (Table 1). Of note, conversion of immunosuppression did not affect plasma HCV-RNA levels, liver-stiffness as assessed by transient elastography or the hepatic fibrosis score FIB-4 (Table 1).

Although PTDM was known in only three study participants at time of inclusion, another subject featured overt diabetes at the initial OGTT.

**Table 1:** Baseline characteristics before and three months after conversion. Baseline characteristics and parameters of renal graft function and liver function before and three months after the conversion from tacrolimus to cyclosporine A. Data are expressed as median (IQR).

Parameter (n = 10)	before conversion	after conversion	p-value
age (years)	51.32 (43.19–57.99)		
time since renal transplantation (years)	8.31 (5.60–12.36)		
number of renal transplant (n)	1.00 (1.00–2.00)		
CDC-PRA, latest (%, n = 9)	4.00 (0–21.50)		
renal replacement therapy (years)	4.211 (2.322–5.561)		
creatinine (μmoL/L)	139.83 (107.09–164.61)	147.80 (123.02–177.89)	0.114
GFR (MDRD, mL/min)	48.22 (40.80–57.51)	42.56 (38.00–56.52)	0.139
urinary protein-creatinine-ratio (mg/g)	182.00 (133.00–1179.50)	471.00 (134.50–1267.00)	0.779
tacrolimus trough level (nmoL/L)	6.283 (5.429–7.351)		
cyclosporine A trough level (nmoL/L)		93.30 (71.22–155.56)	
C2 level (nmoL/L)		344.03 (329.04–645.58)	
body mass index (kg/m²)	26.25 (20.76–29.42)	27.17 (20.59–29.64)	0.779
C-reactive protein (mg/L)	0.950 (0.500–3.025)	1.200 (0.375–3.250)	0.779
TSH (mIU/L)	2.330 (1.750–3.710)	2,620 (1.610–3.840)	0.445
albumin (g/L)	404.0 (390.3–438.3)	421.5 (399.3–436.3)	0.153
total cholesterol (mmoL/L)	4.102 (3.496–4.599)	5.070 (3.818–5.966)	0.028
LDL-C (mmoL/L)	2.198 (1.731–2.655)	2.828 (2.091–3.786)	0.051
HDL-C (mmoL/L)	1.032 (0.890–1.367)	1.019 (0.935–1.393)	0.674
triglycerides (mmoL/L)	1.174 (0.909–1.875)	1.682 (1.211–2.890)	0.047
total bilirubin (μmoL/L)	11.97 (7.49–21.00)	17.10 (9.03–23.22)	0.066
aspartate aminotransferase (U/L)	29.50 (22.75–39.50)	23.00 (20.00–39.50)	0.047
alanine aminotransferase (U/L)	30.00 (24.75–44.75)	22.00 (17.50–25.75)	0.028
gamma-glutamyl transferase (U/L)	54.50 (19.50–141.75)	36.50 (14.50–147.50)	0.139
normotest (%)	114.00 (96.50–134.50)	122.00 (107.50–141.50)	0.123
HCV-PCR (copies/mL)	546000 (351750–2965000)	2560000 (798000–3370000)	0.285
liver stiffness (kPA, n = 8)	6.80 (5.60–13.65)	10.25 (6.15–20.43)	0.463
FIB-4	2.00 (1-60-4.03)	2.25 (1.58–4.20)	0.337

### Gluco-metabolic parameters

**Fig 2:**
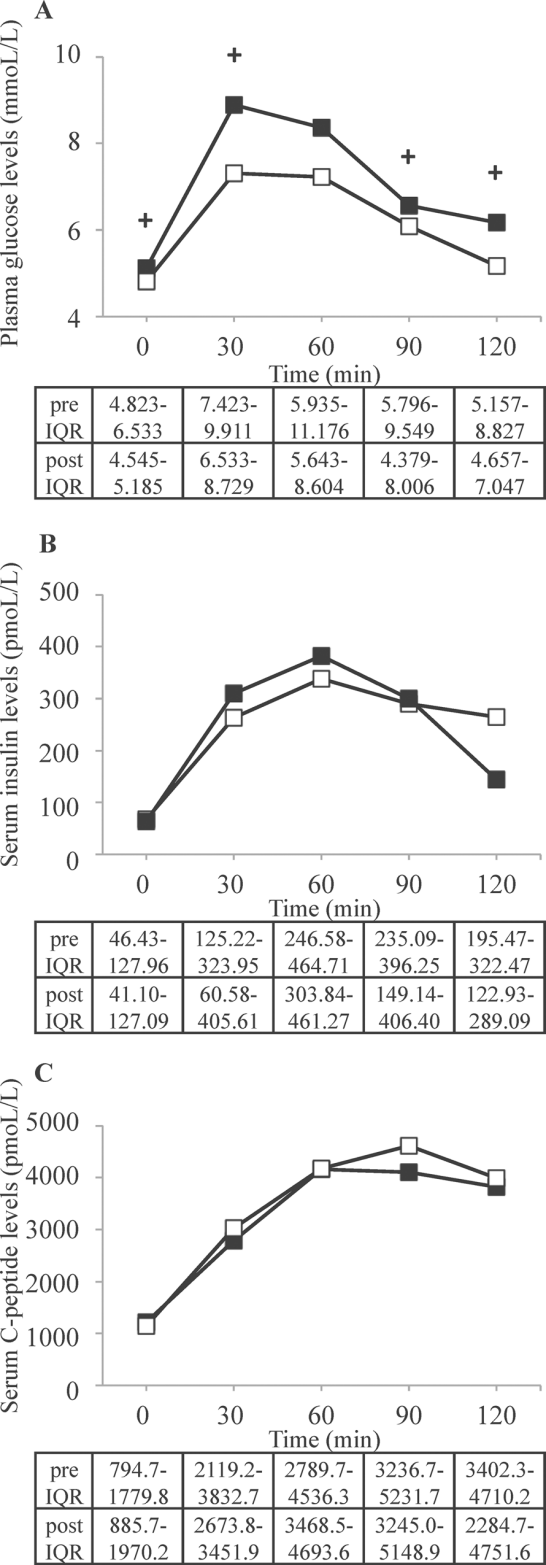
Metabolic parameters before and three months after conversion. Determination of plasma glucose (A), serum insulin (B) and C-peptide (C) levels by OGTTs performed prior to (full symbols) and three months after (blank symbols) the conversion from taxrolimus to cyclosporine A. Data are expressed as median (IQR). + indicates p <0.05 (in detail for plasma glucose levels: p = 0.012 at 0min, p = 0.025 at 30 min, p = 0.059 at 60 min, p = 0.022 at 90 min, p = 0.028 at 120 min).

Following the conversion of CNIs, fasting and challenged plasma glucose levels significantly decreased (Fig 2A), but serum insulin and C-peptide levels were unaltered indicating a favourable and distinct impact of CyA on glucose metabolism (Fig 2B and 2C).

Dynamic insulin sensitivity parameter OGIS increased highly significantly post-conversion (p = 0.005, Table 2) indicating a marked amelioration of dynamic insulin sensitivity. This was also reflected by a significant improvement of the adaptation index (AI, p = 0.017) as marker of beta-cell function and the disposition index (DI, p = 0.017), both evidences of improved overall glucose tolerance (Table 2). First-phase insulin secretion as evaluated by the insulinogenic index tended to improve (p = 0.093, Table 2), while HbA1c, hepatic insulin extraction and QUICKI as marker of fasting (mostly hepatic) insulin sensitivity did not change significantly (Table 2).

**Table 2:** Gluco-metabolic parameters before and three months after conversion. Insulin sensitivity, insulin secretion and other parameters of glucose tolerance before and three months after the conversion from tacrolimus to cyclosporine A. Data are expressed as median (IQR).

Parameter (n = 10, respectively)	before conversion	after conversion	p-value
OGIS (mL/min/m^2^)	422.17 (370.82–441.92)	468.80 (414.27–488.57)	0.005
QUICKI	0.416 (0.402–0.451)	0.435 (0.406–0.458)	0.721
Insulinogenic Index (nmoL/mmoL)	0.064 (0.032–0.106)	0.083 (0.054–0.144)	0.093
Disposition Index	1.303 (0.991–1.574)	1.533 (1.351–1.971)	0.017
Adaptation Index	17.81 (15.04–23.13)	22.78 (18.97–29.22)	0.017
hepatic insulin extraction (%)	78.09 (68.44–82.26)	77.60 (67.01–81.67)	0.646
HbA1c (%)	5.70 (5.05–6.18)	5.30 (5.08–5.80)	0.385

No significant correlations were found between ΔOGIS, ΔDI or ΔAI and changes (Δ) in parameters of renal or hepatic function, lipids, thyroid hormones or BMI (all p>0.05).

**Fig 3:**
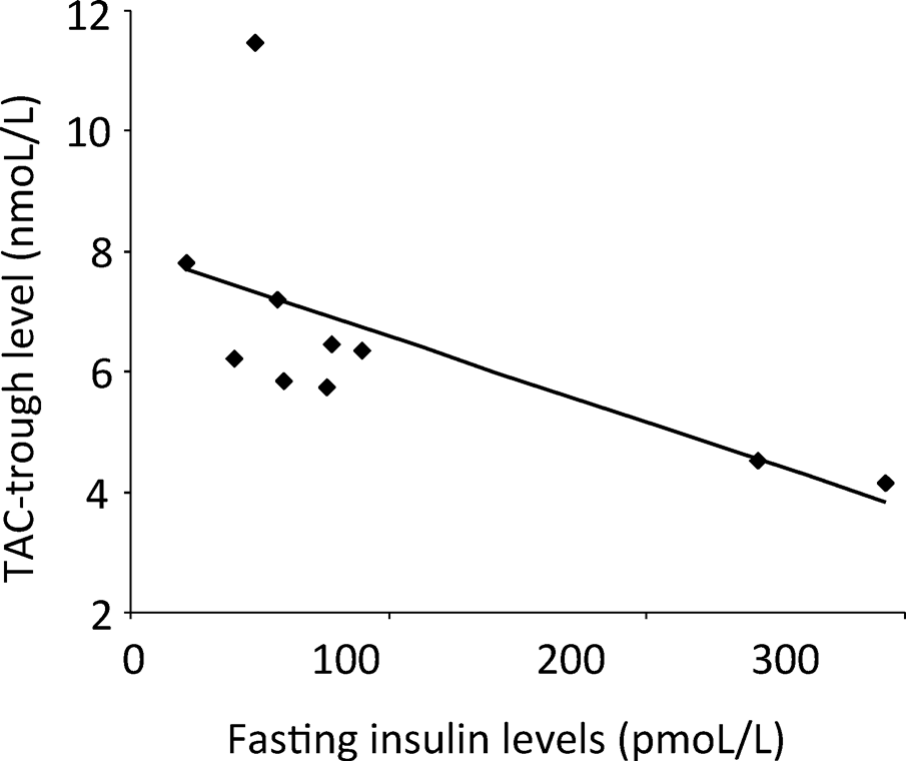
Correlation analyses. Scatter plots showing the correlation between tacrolimus trough levels and fasting insulin levels before conversion from tacrolimus to cyclosporine A.

TAC-trough levels correlated negatively with fasting insulin (r = -0.709, p = 0.022, Fig 3) before conversion, but not with OGIS, AI, DI, IGI or fasting glucose and C-peptide levels (p>0.05, respectively). No significant correlation was observed between subsequent CyA-trough levels or CyA concentrations measured after 2 hours (C_2_-levels) and OGIS, DI, AI, IGI or fasting glucose, insulin and C-peptide levels (all p>0.05).

Additionally, no differences were observed in ΔOGIS (p = 0.352), ΔQUICKI (p = 0.762), ΔDI (p = 0.114), ΔAI (p = 0.610) or ΔIGI (p = 0.610) between subjects with PTDM and those with normal glucose tolerance.

## Discussion

Here we demonstrate that the conversion from TAC to CyA results in a profound reduction of both fasting and challenged plasma glucose concentrations without increasing the pancreatic effort in terms of an additionally aggravated hyperinsulinemia. Of note, the observed beneficial effects were mainly attributable to a highly significant amelioration of dynamic (post-challenge) insulin sensitivity and to a lesser extent to an improvement of first-phase insulin secretion, which together resulted in enhanced overall glucose tolerance in HCV-positive RTRs. It is worth noting also that post-challenge insulin sensitivity mimics the process in post-prandial dynamic conditions, the depiction of which is fundamental for characterizing the metabolic state of a subject.

Most previous studies comparing the diabetogenicity of TAC versus CyA were cross-sectional in design and reported on lower PTDM-rates under CyA- as compared to TAC-treatment [1]. However, the available CNI-conversion studies showed conflicting results: whereas some trials reported an improved fasting glycemia and lower HbA1c levels after the conversion to CyA [1,22,23], others failed to observe significant changes [1,24]. These inconsistencies were attributed to variations in PTDM-definition, diagnostic tools, the dose dependency of TAC-effects and the modulation of the TAC-related diabetogenicity by concomitant immunosuppressive treatment with steroids or MPA [1,25]. Renal function and a uremic environment may also influence CNI-related effects on glucose homeostasis as CNIs were found to improve euglycemic-hyperinsulinemic clamp derived M/I-value in healthy subjects but to decrease insulin sensitivity in patients on hemodialysis [26,27].

As mentioned above, only few studies assessed dynamic/glucose-stimulated parameters of insulin sensitivity and insulin secretion in RTRs. In contrast to our results, recent data from Bulanowski et al. [28] using euglycemic-hyperinsulinemic clamps for the evaluation of dynamic insulin sensitivity showed no changes following the conversion from CyA to TAC. The discrepant results may be explained by differences in concomitant immunosuppressive medication or HCV status. TAC- and CyA-trough levels were similar in both trials, but we included only subjects with MPA and/or steroids as concomitant immunosuppression but not like Bulanowski et al. [28] with azathioprine or sirolimus, which is a well-known modulator of insulin secretion and sensitivity [3]. Further, no information on the HCV status was provided [28]. This, however, is pivotal as HCV infection presumably amplifies TAC-related hyperglycemia [29]. Bloom et al. [29] found significantly higher PTDM-rates among TAC- as compared to CyA-treated RTRs only in the presence of HCV infection. Whereas HCV positivity was associated with a seven-fold increase of PTDM in TAC-treated subjects, HCV-positive CyA-treated RTRs displayed similar PTDM-rates as HCV-negative RTRs. A multivariate regression model revealed a significant interaction between HCV positivity and TAC- but not CyA-treatment [29].

The association between HCV positivity and insulin resistance is well recognized and may involve an HCV-related impairment of insulin signaling, production of proinflammatory cytokines and induction of liver fibrosis resulting in a reduced hepatic and systemic insulin sensitivity and responsiveness [6,7,10]. Of note, insulin resistance, but not impaired insulin secretion or hepatic insulin uptake, was shown to be the predominant feature of HCV-associated glucose intolerance in non-diabetic RTRs [9]. Interestingly, HCV-related insulin resistance confers a resistance to interferon-based anti-HCV treatment and independently promotes the progression of HCV-related hepatic complications including steatosis, fibrosis and hepatocellular carcinoma [10]. Thus, it is intriguing to propose that the amelioration of insulin sensitivity by the conversion of immunosuppression from TAC to CyA slows down the natural course of HCV-related liver disease and improves viral response to anti-HCV treatment as was shown for insulin-sensitizer pioglitazone in patients with HCV genotype 4 [30]. Indeed, CyA but not TAC was recently found to reduce HCV-RNA replication and HCV protein production *in vitro* and *in vivo* [11,12,31]. The main intracellular ligand of CyA, cyclophilin A, which after complexed by CyA inhibits calcineurin, is an essential cofactor for HCV infection and replication, whereas the FK-binding protein-12 as main ligand of TAC exerts no effects on HCV replication [31]. In subjects enrolled in our study, HCV-PCR results, liver stiffness (a marker of hepatic fibrosis and necroinflammation) and liver fibrosis-score Fib-4 remained unchanged three months after the conversion from TAC to CyA. This, however, may be at least in part due to CyA levels lower than necessary to inhibit HCV replication. *In vitro*-experiments have shown that CyA reduces HCV-RNA and -protein production at levels at levels >1000 ng/mL [11]. Such high doses could not be maintained in our study population as serum creatinine and bilirubin increased during the course of the study, an adverse effect which resolved after dose reduction of CyA. Despite the lack of effects on HCV-RNA levels, liver stiffness and the FIB-4 liver fibrosis score, CyA-treatment results in a significant attenuation of dynamic insulin resistance, which suggests that CyA itself, in terms of a substance-related effect, rather than HCV infection accounts for the observed beneficial changes in glucose homeostasis. Considering that chronic HCV infection promotes hepatic and systemic insulin resistance, CyA may be especially advantageous for HCV-positive RTRs and a conversion of immunosuppression from TAC to CyA—from a pathophysiological point of view—a sensible approach to improve post-prandial glycemic control in RTRs with HCV-induced insulin resistance and possibly RTRs with pronounced insulin resistance in general.

However, impaired insulin secretion rather than insulin resistance was suggested to represent the predominant mechanism underlying PTDM [32,33]. A large multi-center study [32] comparing OGTT-derived parameters of glucose tolerance in 1064 RTRs and 1357 non-transplanted subjects found a diminished insulin secretion but an augmented insulin sensitivity in subjects with previous renal transplantation. Adding to an on-going debate about the relative distribution of defects in insulin sensitivity or insulin secretion in PTDM [34–37], the authors of this large survey proposed beta-cell dysfunction as the primary defect in PTDM with a possibly compensatorily increased insulin sensitivity [32]. Indeed, several studies have reported that CNIs impair beta-cell function in terms of decreasing insulin transcription and promoting beta-cell apoptosis *in vitro* [37]. In our study, glucose-stimulated first-phase insulin secretion as assessed by IGI tended to improve and TAC-trough levels correlated negatively with fasting insulin levels. Our findings corroborate previous reports indicating a negative impact of TAC on beta-cell function [25,37] and suggest that the impairment of beta-cell function by TAC exceeds that of CyA, which might represent another beneficial effect of a CNI-conversion besides the marked and predominant amelioration of insulin sensitivity.

HCV is an independent risk factor for the development of PTDM [1,2]. It might be speculated that one reason why HCV promotes PTDM is that the additional impairment of insulin secretion by CNIs to the pre-existing HCV-induced insulin resistance results in a decompensation of glucose homeostasis. Here we show that CyA as compared to TAC exerts beneficial properties on both insulin sensitivity and insulin secretion.

Despite the marked amelioration of glucose tolerance, the conversion from TAC to CyA also elicited adverse side effects including a well-acknowledged hyperlipidemia and a statistically non-significant increase of serum creatinine levels. This, however, is one of the main limitations of our study as the sample size, although adequately powered to observe changes in OGIS, was too small to evaluate safety issues. In clinical practice, numbers of HCV-positive RTRs declined in the last years, affecting only 3.3% (46 of 1394) of all RTRs at our center during the study period. This is in line with other epidemiologic data showing a decreasing number of HCV-positive subjects in European ESRD-population–likely related to improved hygienic precautions during hemodialysis, use of erythropoietin-stimulating agents (rather than RBC transfusions), screening of kidney donors and the development of more efficient anti-HCV therapies including interferon-free regimens of directly acting antivirals [38,39]. Another limitation of our study is that—given the known HCV-related insulin resistance—we cannot exclude that the beneficial gluco-metabolic effects of a TAC-to-CyA-conversion may be less pronounced in HCV-negative RTRs as we did not include a HCV-negative control collective. Although our data suggest that the beneficial gluco-metabolic effects are substance-related, *i*.*e*. improved dynamic insulin sensitivity under CyA- as compared to TAC-treatment, further studies are needed to evaluate whether our findings may also apply to HCV-negative RTRs.

Nonetheless, our findings entail significant clinical implications for the post-transplant management of HCV-positive subjects. Diabetes remains a major cause for ESRD and HCV infection represents a major issue in many parts of the world with a prevalence of up to 68% in subjects with ESRD [40]. Similar to type 1 and type 2 diabetes mellitus in the non-transplanted population, PTDM causes several adverse conditions in RTRs [1,2], Data from the United Renal Data System (USRDS) provided evidence that PTDM increases the risk of graft failure by 63%, the risk of death-censored graft failure by 46% and the risk for mortality by 87% as compared to subjects without diabetes [4]. In addition, PTDM is a strong risk factor for cardiovascular disease, the main cause of mortality in these subjects and RTRs in general. Apart from these rather well-established complications, PTDM might also relate to microvascular disease, an increased rate of infections and events related to hyper- or hypoglycemic episodes [1,2]. Further, with deteriorating transplant-function and frequently existing co-morbidities, like above mentioned cardiovascular disease, therapeutic options are sometimes limited to few oral anti-hyperglycemic agents and anabolic insulin, which might result in an additional worsening of glucose homeostasis due to weight gain. Also the need for pharmaceutical treatment of other cardiovascular risk factors confers an increased risk of drug accumulation and pharmacological interactions with immunosuppressants [3]. To date, immunosuppression for RTRs is routinely conducted by induction with an interleukin-2 receptor antibody and maintenance treatment with steroids, MPA and TAC but not CyA in many transplant centers due to the ELiTE-Symphony study [41]. Considering the HCV-induced insulin resistance, the PTDM-related clinical complications and the presumable HCV-related potentiation of adverse glycemic TAC-effects, our findings on a significant amelioration of glucose-stimulated insulin sensitivity and overall glucose tolerance as well as the improvement of insulin secretion following a TAC-to-CyA-conversion suggest that HCV-positive RTRs may substantially benefit from CyA-based immunosuppressive regimens.

## Supporting Information

S1 TREND Checklist The filled TREND checklist for this study. (PDF)

S1 Protocol The study protocol for this trial. (DOC)

## Abbreviations

AI: adaptation index
AUC: area-under-the-curve
CDC-PRA: complement-dependent cytotoxicity—panel reactive antibodies
CNI: calcineurin-inhibitor
CyA: cyclosporine A
DI: disposition index
eGFR: estimated glomerular filtration rate
ELiTE-Symphony study: Efficacy Limiting Toxicity Elimination-Symphony
ESRD: end-stage renal disease
HCV: hepatitis C-virus
HIE: hepatic insulin extraction
HOMA-IR: homeostasis model assessment of insulin resistance
IGI: insulinogenic index
OGIS: oral glucose insulin sensitivity-index
OGTT: oral glucose tolerance test
PTDM: post-transplant diabetes mellitus
QUICKI: quantitative insulin sensitivity check-index
RTRs: renal transplant recipients
TAC: tacrolimus
TIS: total insulin secretion
