# Supplementary material for: Conversion from Tacrolimus to Cyclosporine A Improves Glucose Tolerance in HCV-Positive Renal Transplant Recipients
Source: PLoS One. 2016 Jan 6;11(1):e0145319. doi: 10.1371/journal.pone.0145319 (PMC4703220; doi:10.1371/journal.pone.0145319)
Supplement: S1 Protocol — (DOC) [file pone.0145319.s002.doc]

Clinical Study Protocol

**Hepatitis C in renal transplant recipients –**

**Safety and efficacy of a conversion of immunosuppression to high-dose cyclosporine A and its impact on HCV-replication, parameters of liver function and glucose tolerance. An open label trial.**

Version 1.0 / 01-Feb-2011

Confidentiality Statement

The information contained in this document, especially unpublished data, is the property of the sponsor of this study, Medical University of Vienna, Department of Internal Medicine III, Division of Nephrology and Dialysis. It is therefore provided to you in confidence as an investigator, potential investigator, or consultant, for review by you, your staff, and an Independent Ethics Committee or Institutional Review Board. It is understood that this information will not be disclosed to others without written authorization from the Principal Investigator except to the extent necessary to obtain informed consent from those persons to whom the study drug may be administered.

| **Test drug (IMP) and Pharmaceutical Company** | Cyclosporine A, Novartis Pharma GmBH |
| --- | --- |
| **Protocol authors** | Univ.-Doz. Dr. Marcus Säemann, Dr. Ammon Handisurya |
| **Principal investigators** | Univ.-Prof. Dr. Alice Schmidt |
| **Document type** | Clinical study protocol |
| **Study phase** | IV |
| **Document status** | complete |
| **Date** | 01-Feb-2011 |
| **Number of pages** | 50 |

| SPONSOR, INVESTIGATOR, MONITOR AND SIGNATURES  | Principal investigator (AMG §§ 2a, 35,36) | Univ.-Prof. Dr. Alice Schmidt, Department of Internal Medicine III, Medical University of Vienna, Austria | | --- | --- | |  | _________________ ___________  Signature Date | | Sponsor/OEL (AMG §§ 2a, 35,36) | Univ.-Prof. DDr. Walter H. Hörl, Department of Internal Medicine III, Division of Nephrology and Dialysis, Medical University of Vienna, Austria | |  | _________________ ___________  Signature Date | | Representative of CRO (AMG §§ 2a, 35,36) | Univ.-Prof. Dr. Anna-Christine Hauser, Department of Internal Medicine III, Division of Nephrology and Dialysis, Medical University of Vienna, Austria | |  | _________________ ___________  Signature Date | |  |  | |  |  |   Associated Departments: Department of Internal Medicine III, Division of Gastroenterology and Hepatology, Medical University of Vienna, Austria  Clinical Trials Centers: Department of Internal Medicine III, Division of Nephrology and Dialysis, Medical University of Vienna, Austria |
| --- | --- | --- | --- | --- | --- | --- | --- | --- | --- | --- | --- | --- | --- | --- | --- | --- |

#

# 2 Protocol Synopsis

| TITLE | Hepatitis C in renal transplant recipients – The impact of cyclosporine A on HCV-replication, parameters of liver function and glucose tolerance | | | | | |
| --- | --- | --- | --- | --- | --- | --- |
| ACRONYM |  | | | | | |
| OBJECTIVES | **Primary Objective**   - To assess the impact of a conversion to a cyclosporine A-based immunosuppressive regimen on HCV-replication and parameters of liver function in renal transplant recipients   **Secondary Objectives**   - To assess the impact of a conversion to a cyclosporine A-based immunosuppressive regimen on parameters of insulin sensitivity and insulin secretion in renal transplant recipients | | | | | |
| DESIGN / PHASE | Prospective, mono-center, open, phase IV study. | | | | | |
| STUDY PLANNED DURATION | First patient  First visit | *3Q/*  *2011* | Last patient  **First visit** | *2Q/*  *2012* | **Last patient**  **Last visit** | *3Q/*  *2012* |
| CENTER(S) / COUNTRY(IES) | One center in one country.  Austria | | | | | |
| PATIENTS / GROUPS | 50 patients in one group | | | | | |
| INCLUSION CRITERIA | - .Written informed consent - .Prior renal transplantation with current immunosuppression other than cyclosporine A - Hepatitis C-infection - Age 18-70 years | | | | | |
| EXCLUSION CRITERIA | - Current hemodialysis or peritoneal dialysis - Pregnancy or breastfeeding - Known contraindications for / intolerability of calcineurin-inhibitors | | | | | |
| STUDY PERIODS |  | | | | | |
| INVESTIGATIONAL DRUG | **MED 1: Cyclosporine A**  initial dose: 10 mg/kg body weight/day  target dose: serum concentration 2 hours after administration 500-1000 ng/ml | | | | | |
| COMPARATIVE DRUG | Not applicable | | | | | |
| CONCOMITANT MEDICATION | **Allowed** | | | | | |
| EFFICACY ENDPOINTS | *HCV-RNA, l*iver transaminases, fibroScan-Indices, IL28-SNP, vitamin D, parameters of insulin sensitivity and insulin secretion, plasma adipokine levels | | | | | |
| TOLERABILITY / SAFETY ENDPOINTS | Glomerular filtration rate | | | | | |
| PHARMACOKINETIC / PHARMACODYNAMIC ENDPOINTS |  | | | | | |
| QUALITY OF LIFE / PHARMACOECONOMIC  ENDPOINTS |  | | | | | |
| STATISTICAL METHODOLOGY | - **Primary Endpoint:** HCV-RNA - **Null and alternative hypotheses:**   **H_0_** conversion from a tacrolimus- to a cyclosporine A-based immunosuppression has no effects on HCV-replication  **H_1_**: conversion from a tacrolimus- to a cyclosporine A-based immunosuppression attenuates HCV-replication by one decimal power (one unit of the decadic logarithm) after 12 weeks  **Type-I and -II errors - power.**  α = 5 %  ß = 90 %  .   - **Statistical methodology**   .Paired t-test   - **Sample size calculation.**   A sample size of 26 will have 90% power to detect a difference in means of 1, assuming a standard deviation of differences of 1,5, using a paired t-test with a 0,050 two-sided significance level. Considering a drop-out rate of 10 percent results in a sample size of about 30 patients. If more patients are willing to participate in the study, up to 50 patients will be recruited. In this case more refined mean differences can be detected or a possible higher standard deviation of differences can be accounted for.   - **Main analysis set** - **Other endpoints** | | | | | |
| STUDY EXTENSION | *.* | | | | | |

# LIST OF ABBREVATIONS

AFP … alpha fetoprotein

CNI … calcineurin-inhibitor

CsA … cyclosporine A

CyPA … cyclophilin A

ESRD … endstage renal disease

FFA … free fatty acids

HCV … hepatitis C virus

HIE … hepatic insulin extraction

HLA … human leukocyte antigen

HOMA-IR … homeostasis model assessment of insulin resistance

Il … interleukin

IP-10 … interferon gamma-inducible protein-10

INF … interferon

KDIGO … kidney disease: improved global outcomes

mTOR … mammalian target of rapamycin

NF-AT … nuclear factor of activated T-cells

NIH … National Institute of Health

NODAT … new onset diabetes after transplantation

OGIS … oral glucose insulin sensitivity (index)

OGTT … oral glucose tolerance test

PTDM … posttransplant diabetes mellitus

SNP … single nucleotide polymorphism

SVR … sustained virologic response

TIS … total insulin secretion

USRDS … United States Renal Data system

# TABLE OF CONTENTS

[1 SPONSOR, INVESTIGATOR, MONITOR AND SIGNATURES 3](#__RefHeading___Toc282441419)

[2 PROTOCOL SYNOPSIS 4](#__RefHeading___Toc282441420)

[3 LIST OF ABBREVATIONS 5](#__RefHeading___Toc282441421)

[TABLE OF CONTENTS 7](#__RefHeading___Toc282441422)

[4 INTRODUCTION 11](#__RefHeading___Toc282441423)

[4.1 Background information 11](#__RefHeading___Toc282441424)

[4.2 Study rationale 14](#__RefHeading___Toc282441425)

[5 STUDY OBJECTIVES 15](#__RefHeading___Toc282441426)

[5.1 Primary Objective 15](#__RefHeading___Toc282441427)

[5.2 Secondary Objectives 15](#__RefHeading___Toc282441428)

[6 STUDY DESIGN 15](#__RefHeading___Toc282441429)

[6.1 Study population 15](#__RefHeading___Toc282441430)

[6.1.1 Subject population 19](#__RefHeading___Toc282441431)

[6.1.2 Inclusion criteria 19](#__RefHeading___Toc282441432)

[6.1.3 Exclusion criteria 19](#__RefHeading___Toc282441433)

[6.1.4 Females of childbearing age 19](#__RefHeading___Toc282441434)

[6.1.5 Study duration 20](#__RefHeading___Toc282441435)

[6.1.6 Withdrawal and replacement of subjects 20](#__RefHeading___Toc282441436)

[6.1.7 Premature termination of the study 21](#__RefHeading___Toc282441437)

[7 METHODOLOGY 21](#__RefHeading___Toc282441438)

[7.1 Prestudy screening investigation 21](#__RefHeading___Toc282441439)

[7.2 Dose selection, treatment duration and modification 21](#__RefHeading___Toc282441440)

[7.3 Study medication 21](#__RefHeading___Toc282441441)

[7.3.1 Dosage and administration 22](#__RefHeading___Toc282441443)

[7.3.2 Study-drug up- and down titration 22](#__RefHeading___Toc282441444)

[7.3.3 Study drug interruption or discontinuation 22](#__RefHeading___Toc282441445)

[7.3.4 Study-drug delivery, packaging and labeling 23](#__RefHeading___Toc282441446)

[7.3.5 Concomitant medication 23](#__RefHeading___Toc282441447)

[7.3.6 Interactions, reverse reactions and side effect of IMP: 24](#__RefHeading___Toc282441448)

[7.3.7 Randomization and stratification: 25](#__RefHeading___Toc282441449)

[7.3.8 Blinding 25](#__RefHeading___Toc282441450)

[7.4 Study procedures 26](#__RefHeading___Toc282441451)

[7.4.1 Description of study procedures 26](#__RefHeading___Toc282441452)

[8 SAFETY DEFINITIONS AND REPORTING REQUIREMENTS 30](#__RefHeading___Toc282441453)

[8.1 Averse events (AEs) 30](#__RefHeading___Toc282441454)

[8. 1. 1 Summary of known and potential risks of the study drug 30](#__RefHeading___Toc282441455)

[8. 1.2 Definition of adverse events 31](#__RefHeading___Toc282441456)

[8. 2 Serious adverse events (SAEs) 32](#__RefHeading___Toc282441457)

[8. 2. 1 Hospitalization – Prolongation of existing hospitalization 32](#__RefHeading___Toc282441458)

[8. 2. 3 Suspected unexpected serious adverse reactions (SUSARs) 33](#__RefHeading___Toc282441459)

[8. 3 Severity of adverse events 34](#__RefHeading___Toc282441460)

[8. 5 Reporting procedures 35](#__RefHeading___Toc282441461)

[8. 5. 1 Reporting procedures for SAEs 36](#__RefHeading___Toc282441462)

[8. 5. 2 Reporting procedures for SUSARs 36](#__RefHeading___Toc282441463)

[8. 5. 3 Annual Safety Report 37](#__RefHeading___Toc282441464)

[9 FOLLOW UP 37](#__RefHeading___Toc282441465)

[9.1 Follow-up of study participants including follow-up of adverse vents 37](#__RefHeading___Toc282441466)

[9.2 Treatment after end of study 37](#__RefHeading___Toc282441467)

[10 STATISTICAL METHODOLOGY AND ANALYSIS 38](#__RefHeading___Toc282441468)

[10.1 Analysis sets 38](#__RefHeading___Toc282441469)

[10.2 Sample size considerations 38](#__RefHeading___Toc282441470)

[10.3 Relevant protocol deviations 38](#__RefHeading___Toc282441471)

[10. 4 Endpoints analysis 39](#__RefHeading___Toc282441473)

[10. 4. 1 Primary endpoint analysis 39](#__RefHeading___Toc282441474)

[10. 4. 2 Secondary endpoint analysis 39](#__RefHeading___Toc282441475)

[10. 4. 3 Safety and tolerability endpoints 39](#__RefHeading___Toc282441476)

[10. 4. 4 Baseline parameters and concomitant medications 39](#__RefHeading___Toc282441477)

[10. 5 Exploratory analyses 39](#__RefHeading___Toc282441478)

[10. 6 Interim analysis 39](#__RefHeading___Toc282441479)

[10. 7 Software program(s) 39](#__RefHeading___Toc282441480)

[11 DOCUMENTATION AND DATA MANAGEMENT 39](#__RefHeading___Toc282441481)

[11. 1 Documentation of study results 39](#__RefHeading___Toc282441482)

[11. 1. 1 Case report form (CRF) 40](#__RefHeading___Toc282441483)

[11. 2 Safekeeping 40](#__RefHeading___Toc282441484)

[11. 3 Monitoring 41](#__RefHeading___Toc282441485)

[11.5 Reporting and Publication 42](#__RefHeading___Toc282441486)

[12 ETHICAL AND LEGAL ASPECTS 42](#__RefHeading___Toc282441488)

[12. 1 Informed consent of subjects 43](#__RefHeading___Toc282441489)

[12. 2 Acknowledgement / approval of the study 43](#__RefHeading___Toc282441490)

[12. 2 .1 Changes in the Conduct of the Study 44](#__RefHeading___Toc282441491)

[12.2.1.1 Protocol amendments 44](#__RefHeading___Toc282441492)

[12.2.1.2 Study Termination 44](#__RefHeading___Toc282441493)

[12. 3 Finance and insurance 44](#__RefHeading___Toc282441494)

[12. 4 Confidentiality 44](#__RefHeading___Toc282441495)

[12. 5 Ethics and Good Clinical Practice (GCP) 45](#__RefHeading___Toc282441497)

[13 APPENDICES 46](#__RefHeading___Toc282441498)

[14 REFERENCES 46](#__RefHeading___Toc282441499)

Table 1: Visit and assessment schedule

| **PERIODS** | **Name** | **SCREENING** | **TREATMENT** | | | | **Premature EOS** |
| --- | --- | --- | --- | --- | --- | --- | --- |
|  | **Duration** |  | ***12 weeks*** | | | |  |
| **VISITS** | **Number** | ***1*** | ***2*** | ***3*** | ***4*** | ***5*** |  |
|  | **Name** | ***Screening*** | ***Conversion*** | ***Control 1*** | ***Control 2*** | ***End of Study*** |  |
|  | **Time** | ***Week -1*** | ***Day 0*** | ***Week 4 (± 2 days)*** | ***Week 8 (± 2 days)*** | ***Week 12 (± 2 days)*** |  |
| **Informed Consent** | | x |  |  |  |  |  |
| **Inclusion / Exclusion Criteria** | | x |  |  |  |  |  |
| **Medical History** | | x |  |  |  |  |  |
| **Concomitant/change in medication** | |  | x | x | x | x | x |
| **Physical Examination** | | x |  |  |  | x | x |
| **Body weight and height** | | x | x | x | x | x | x |
| **Vital Signs (BP, PR)** | | x | x | x | x | x | x |
| **12-lead ECG** | | x |  |  |  |  |  |
| **Laboratory Tests** | | x | x | x | x | x | x |
| **Transient Elastography** | |  | x |  |  | x |  |
| **Pregnancy Test** | | x | x | x | x | x |  |
| **Oral Glucose Tolerance Test** | |  | x |  |  | x |  |
| **Study Drug Accountabililty Check** | |  | x | x | x | x | x |
| **Adverse Events** | |  | x | x | x | x | x |
| **Serious Adverse Events** | |  | x | x | x | x | x |

# INTRODUCTION

## Background information

*4.1.1. Renal transplantation and prevention of allograft rejection*

The prevalence of endstage renal disease (ESRD) is increasing worldwide. The final treatment option for ESRD is renal transplantation, which improves quality of life and prolonges survival relative to dialysis. Following successful transplantation lifelong immunosuppression, however, is compulsory to prevent allograft rejection. Pharmaceutical treatment options, thereby, include corticosteroids, calcineurin-inhibitors (CNI) including cyclosporine A (CsA) and tacrolimus (FK506) as well as antimetabolites mycophenolat mofetil/mycophenolat-sodium (MMF) and mammalian target of rapamycin (mTOR)-inhibitors.

*4.1.2. Hepatitis C in renal transplant recipients*

Hepatitic C virus (HCV)-infection affects approximately 2.5% of the world population [1]. Chronic HCV infection is known to promote the development of liver fibrosis / cirrhosis with associated complications including portal hypertension, hepatic decompensation and hepatocellular carcinoma [2].

Patients, who are anti-HCV positive prior to transplantation, have a significantly higher risk of posttransplant liver disease (RR 5.0) including chronic active hepatitis and fibrosing cholestatic hepatitis [3, 4]. In addition, renal transplantation is associated with an increased HCV-proliferation rate, resulting in a 1.8 to 30.3-fold increase in serum virus titer [4]. However, the virusload does not directly relate to disease activity, i.e. no association with the degree of histological changes on liver biopsy or with liver transaminase patterns [4]. With regard to the impact of pre-transplant anti-HCV on patient survival literature is inconclusive. Data of the New England Organ Bank [4] has shown a 3.3-fold higher risk of death and a 9.9-fold higher risk of death due to sepsis in subjects with anti-HCV prior to renal transplantation. In line, a meta-analysis [5] including 6365 anti-HCV positive patients reported on an increased risk for death (RR 1.79, CI 1-57-2.03) and allograft failure (RR 1.56, CI 1.35-1.80). The attenuated allograft survival might in part be explained by the development of HCV-associated renal disease in kidney recipients. The most common manifestations are posttransplant proteinuria, *de novo* membrano-proliferative glomerulonephritis and membranous nephropathy [6-8]. Additionally, HCV-infection represents a strong risk factor for the development of post-transplant diabetes [9].

For therapy, the administration of interferon alpha (INFα) in combination with ribavirin has been approved for both initial treatment and treatment of a chronic hepatitis C-relapse in subjects without renal disease [10]. In addition to its antiviral activity, effects of INFα, however, include antiproliferative and immunomodulatory properties such as induction of cytokine gene expression, increased expression of HLA antigens and enhanced activity of natural killer cells, cytotoxic T-cells and monocytes [11]. Thereby, INFα has been demonstrated to significantly increase the risk of a subsequent allograft rejection [12]. Thus, the NIH Concensus Statement [13] lists renal transplantation as a relative contraindication for INFα. Consistently, the KDIGO guidelines [14] recommend INFα only in anti-HCV positive renal transplant recipients with fibrosing cholestatic hepatitis or life-threatening vasculitis. With regard to ribavirin, clearance is impaired in subjects with renal dysfunction and, thus, administration is not recommended, if the creatinine clearance is lower than 50 ml/min. The efficacy of ribavirin alone in patients, who cannot be treated with INFα, is controversial and data are limited [15, 16].

With regard to prognostic factors for a sustained virologic response (SVR) high interferon gamma-inducible protein-10 (IP-10) levels prior to the initiation of antiviral treatment were found to correlate with hepatitis C virusload, hepatic inflammation and liver fibrosis and might predict viral response following IFN-therapy [17-19]. In addition, rates of early and SVR were higher in HCV-positive carriers of the interleukin 28-single nucleotide polymorphisms (IL28-SNPs) rs12979860 C/C and rs8099917 T/T [20-22].

*4.1.3. Hepatitis C virus and cyclosporin A*

Interestingly, calcineurin-inhibitor CsA, a common agent in the immunosuppressive regimen following solid organ transplantation, was found to suppress HCV-RNA replication and HCV protein production *in vitro* and *in vivo* [23-26]. The main intracellular ligand of CsA is cyclophilin A (CyPA), which belongs to the family of cyclophilins, cellular enzymes with peptidyl-prolyl isomerise activity. The CsA-CyPA-complex thereafter binds and inhibits calcineurin, which dephosphorylates NF-AT thereby allowing recruitment to the interleukin-2 promoter and consequently successful T-cell activation. Recently, CyPA was shown to be an essential cofactor for hepatitis C virus infection and replication [27].

The extent of HCV-genome suppression and the decrease of HCV-proteins following the *in vitro*-administration of CsA were comparable to that of INFα [26]. CsA exerts this peculiar anti-HCV-activity independent of its immunosuppressive activity as non-immunsuppressive cyclophilin-inhibitors Debio 035, NIM811 and SCY-635 also attenuate HCV RNA and protein production [28-30]. *In vitro* experiments have shown that CsA decreases HCV RNA levels by 2 logs at concentrations >1000 ng/ml [31]. Tacrolimus, another calcineurin-inhibitor, which binds to FK-binding protein-12 had no discernible effects on HCV replication [25]. As a proof of principle Inoue K. et al. [32] demonstrated that in non-transplant patients INFα-therapy combined with CsA was more efficient than INFα alone and resulted in higher virological and biochemical response rates, especially in HCV genotype 1, i.e. reduction of virusload and liver transaminases. Previous studies in liver transplant recipients have suggested a beneficial effect of CsA on liver function, graft survival and hepatitis C recurrence [33, 34]. Data on the influence of CsA on HCV-replication in subjects with prior renal transplantation is limited. Renal transplant recipients on CsA were characterized by similar serum alanine aminotransferase levels pre- and posttransplant, whereas a significant increase was observed in subjects on tacrolimus [35].

*4.1.4. New-onset diabetes mellitus after transplantation*

“New-onset diabetes mellitus after transplantation” (NODAT), also termed as “post-transplant diabetes mellitus” (PTDM), is a rather common complication following solid organ transplantation with an incidence of 2-50% [36, 37]. Recent observational studies have found NODAT-rates to have increased during the last three decades. Similar to type 1 and type 2 diabetes mellitus NODAT is associated with cardiovascular disease and, further, promotes graft failure (RR 1.63, p<0.0001) and mortality in kidney recipients [37-40]. Thus, NODAT also confers a substantial economic burden to health care providers causing additional costs of approx. US$21.500 per patient by two years post-transplant [41]. Several risk factors for the development of NODAT have been identified including higher age, increased body mass-index, impaired fasting glucose and also hepatitis C infection [9, 42]. In the USRDS registry [43], the one year incidence of NODAT in pretransplant HCV-positive patients was 25.6% as compared to 15.4% in HCV-negative patients. A recent meta-analysis [9] on 2502 renal transplant recipients found an adjusted odds ratio for NODAT of 3.97 (95% CI 1.83-8.61).

The precise pathophysiological mechanisms underlying NODAT, especially the permissive influence of hepatitis C, however remain to be elucidated. Previously, NODAT has been suggested to comprise a combination of both insulin resistance and beta-cell failure in addition to increased insulin clearance following restored kidney function [37]. Evidence suggests that immunosuppressive agents account for the vast majority of NODAT cases [37, 42]. Corticosteroid-induced glucose intolerance is a well-known entity characterized, among others, by increased hepatic gluconeogenesis, reduced glucose utilization and decreased binding of insulin to its receptor [37, 42]. Similarily, CNIs CsA and tacrolimus have been found to negatively influence glucose tolerance in experimental and clinical studies. The mode of action is unknown but might include a pancreatic islet cell toxicity with consecutively reduced insulin secretion as well as the induction of peripheral insulin resistance [37, 42]. In contrast, antiproliferative agents MMF and azathioprine have been suggested to reduce the risk of NODAT [37, 42]. However, it is unclear whether the amelioration of glucose tolerance is induced by MMF itself or rather due to the reduction of concomitant CNIs und steroids [37, 42].

*4.1.5. Vitamin D in chronic liver disease and glucose intolerance*

Apart from its role in calcium-phosphate-metabolism, Vitamin D exerts pleiotropic functions including regulation of innate immunity. Vitamin D from skin and dietary intake is hydroxylated in liver (25-OH-Vitamin D) and kidneys to its active form 1,25-OH-Vitamin D. Consequently, diseases of liver and/or kidneys result in an abnormal vitamin D metabolism / production. Interestingly, low vitamin D levels were shown to relate to increased levels of hepatic fibrosis and also to predict a reduced responsiveness to IFN-based therapy in subjects with chronic hepatitis C [44, 45]. Consistently, Vitamin D supplementation improves the probability of achieving a SVR in these subjects, i.e. improves the response to antiviral treatment [46]. In addition, vitamin D might also be implicated in the regulation of glucose homeostasis. Previously, hypovitaminosis D was found to be associated with insulin resistance and pancreatic beta-cell dysfunction in subjects at risk for type 2 diabetes and to predict future glucose intolerance [47, 48].

## Study rationale

Hepatitis C virus infection is known to promote the development of liver fibrosis / cirrhosis with associated complications including portal hypertension, hepatic decompensation and hepatocellular carcinoma, thereby reducing overall patient survival. Especially in renal transplant recipients, hepatitis C virus infection is significantly associated with a reduced graft survival, increased mortality and a higher incidence of NODAT. Following solid organ transplantation, immunosuppression is compulsory to prevent graft rejection. To date, immunosuppressive regimens include calcineurin-inhibitors such as CsA or tacrolimus. In contrast to tacrolimus, CsA was found to reduce virus-replication *in vitro* and *in vivo* in liver transplant recipients. Despite these encouraging data, the majority of patients with prior renal transplantation take tacrolimus instead of CsA. In addition, tacrolimus has been reported to attenuate glucose tolerance in solid organ recipients.

# STUDY OBJECTIVES

## Primary Objective

The primary objective of the present study is to evaluate whether the conversion from a tacrolimus- to a CsA-based immunosuppression attenuates HCV-replication and other parameters of HCV disease activity at 2h-CsA-serum levels of 500-1000 ng/ml. Primary outcome parameter will be HCV-RNA and secondary parameters will be alanine transaminase (ALT), aspartate transaminase (AST) and gamma-glutamyltransferase (gGT).

## Secondary Objectives

Further, the impact of a conversion from a tacrolimus- to a CsA-based immunosuppression on parameters of insulin sensitivity and insulin secretion will be evaluated. Outcome parameters include the homeostasis model assessment of insulin resistance (HOMA-IR) index and the oral glucose insulin sensitivity (OGIS) index as parameters of basal and dynamic insulin sensitivity as well as the disposition and the adaptation index as parameters of beta-cell function and overall glucose tolerance (*see 7.4.1.3*).

# Study design

This is a prospective, single-center, open study with a study duration of 13 weeks per subject. Subjects, who are considered eligible for the participation in the present study and who have given written informed consent will be included (n=50; *inclusion and exclusion criteria see 6.1.*).

At visit 2 (= *conversion visit, see fig. 1*) immunosuppression will be converted from a tacrolimus- to a CsA-based regimen.

To evaluate changes in serologic parameters of liver function and HCV activity, blood samples for the measurement of HCV-RNA, liver transaminases, 25OH-Vitamin D and 1,25OH-Vitamin D will be obtained prior to (*visit 2*) as well as 4, 8 and 12 weeks (*visits 3-5*) following the conversion of immunosuppression. In addition, IP-10, rheuma factors, cryoglobulins and alpha-fetoprotein (AFP) will be measured at visits 2 and 5.

At baseline (*visit 2*) HCV genotype and IL28-SNP will be identified in all study participants. To evaluate the grade of liver fibrosis/cirrhosis, liver stiffness will be assessed by non-invasive transient elastography at visits 2 and 5 (*see fig. 1*).

To evaluate effects on parameters of insulin sensitivity and insulin secretion 2h-75g-oral glucose tolerance test (OGTT) will be performed at visit 2 (prior to the conversion) and at visit 5 (12 weeks after the conversion) at fasting state.

*5.1.2.1. Visit 1 (Screening Visit, week -1)*

After having given written informed consent, subjects who are considered eligible for the participation in the present study will undergo following procedures:

- Medical history

- Physical examination

- Vital signs (blood pressure, heart frequency), weight and height

- 12-lead ECG

*5.1.2.2. Visit 2 (Conversion Visit, day 0)*

All study participants will undergo following procedures:

- Measurement of HCV genotype, HCV-RNA, liver transaminases, cryoglobulins, AFP, rheuma

factors, 25OH-Vitamin D, 1,25OH-Vitamin D, IP-10, IL28-SNP

- Oral glucose tolerance test

- Transient elastography

- Conversion of immunosuppression from a tacrolimus- to a CsA-based regimen

*5.1.2.3. Visits 3-4 (weeks 4 and 8)*

Visits 3 and 4 will be scheduled 4 and 8 weeks after visit 2. All study participants will undergo following procedures:

- Measurement of HCV-RNA, liver transaminases, 25OH-Vitamin D, 1,25OH-Vitamin D

*5.1.2.4. Visit 5 (End of Study, week 12)*

Visit 5 will be scheduled 12 weeks after visit 2. All study participants will undergo following procedures:

- Measurement of HCV-RNA, liver transaminases, cryoglobulins, AFP, rheuma factors, 25OH-Vitamin D, 1,25OH-Vitamin D, IP-10

- Transient elastography

- Oral glucose tolerance test

Figure 1. Flow Chart


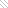


**V1**

**V2**

**V3**

**V4**

**V5**

**Renal**

**Transplantation**

*12 weeks*

*Conversion visit*

*End of Study*

*Screening visit*


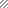


Cyclosporine A

Tacrolimus

*Table 1*. Biochemical measurements

| **Time**  **Visit Week** | | **Parameters** |
| --- | --- | --- |
| Visit 1, Premature End of Study | -1 | potassium, sodium, chloride, calcium, phosphate, magnesium,  creatinine, urea, uric acid, triglycerides, total cholesterol, HDL-C, LDL-C, total bilirubin, total alkaline phosphatase, ALT, AST, GGT, cholinesterase, LDH, CK, albumin, CRP  blood cell count, clotting tests |
| Visit 2 | 0 | potassium, sodium, chloride, calcium, phosphate, magnesium,  creatinine, urea, uric acid, triglycerides, total cholesterol, HDL-C, LDL-C, total bilirubin, total alkaline phosphatase, ALT, AST, GGT, cholinesterase, LDH, CK, albumin, CRP  blood cell count, clotting tests  oral glucose tolerance test, HbA1c, glycated albumin, plasma adipokine levels, 24-urine  TSH, 25OH-vitamin D, 1,25OH-vitamin D, alpha-fetoprotein, IP-10, cryoglobulins, rheuma factors  HCV-RNA  IL28-SNP |
| Visits  3-4 | 4, 8 | potassium, sodium, chloride, calcium, phosphate, magnesium,  creatinine, urea, uric acid, triglycerides, total cholesterol, HDL-C, LDL-C, total bilirubin, total alkaline phosphatase, ALT, AST, GGT, cholinesterase, LDH, CK, albumin, CRP  blood cell count, clotting tests, 24-urine  TSH, 25OH-vitamin D, 1,25OH-vitamin D  serum 2h-CsA-levels  HCV-RNA |
| Visit 5 | 12 | potassium, sodium, chloride, calcium, phosphate, magnesium,  creatinine, urea, uric acid, triglycerides, total cholesterol, HDL-C, LDL-C, total bilirubin, total alkaline phosphatase, ALT, AST, GGT, cholinesterase, LDH, CK, albumin, CRP  blood cell count, clotting tests  oral glucose tolerance test, HbA1c, glycated albumin, plasma adipokine levels, 24-urine  TSH, 25OH-vitamin D, 1,25OH-vitamin D, alpha-fetoprotein, IP-10, cryoglobulins, rheuma factors  serum 2h-CsA-levels  HCV-RNA |

## Study population

### Subject population

All study participants will be recruited among the patients with prior renal transplantation, who attend the Department of Internal Medicine III, Division of Nephrology and Dialysis, Medical University of Vienna, for standard care. According to a recent evaluation, the current number of renal transplant recipients with known Hepatitis C-infection at the outpatient department of the Division of Nephrology is estimated at 50 patients, all considered eligible for study inclusion.

### Inclusion criteria

- Written informed consent

- Prior renal transplantation with a current tacrolimus-based immunosuppressive regimen

- Hepatitis C-infection

- Age 18-70 years

### Exclusion criteria

- Current hemodialysis or peritoneal dialysis

- Pregnancy or breastfeeding

- Known CsA-intolerance

### Females of childbearing age

Women with childbearing potential are considered eligible for the participation in the present trial as immunosuppression is compulsory for renal transplant recipients and CsA first-line treatment in pregnant subjects. Due to the potential effects of a pregnancy on parameters of renal and/or hepatic function, women who are pregnant at the screening visit or become pregnant during the trial will be excluded or withdrawn from the further participation in the present study.

In addition, it should be mentioned that at our center female renal transplant recipients are advised to use oral contraceptives and to plan their pregnancy, i.e. optimization of blood pressure, adjustment of immunosuppressive agents to CsA and of concomitant medication prior to conception.

### Study duration

13 weeks per study participant

### Withdrawal and replacement of subjects

Criteria for withdrawal

Subjects may prematurely discontinue from the study at any time. Premature discontinuation from the study is to be understood when the subject did not undergo EOS examination and / or all pivotal assessments during the study, i.e. three complete dosage-time profiles.

Subjects must be withdrawn under the following circumstances:

- at their own request
- if the investigator feels it would not be in the best interest of the subject to continue
- if the subject violates conditions laid out in the consent form / information sheet or disregards instructions by the study personal

In all cases, the reason why subjects are withdrawn must be recorded in detail in the CRF and in the subject’s medical records. Should the study be discontinued prematurely, all study materials (complete, partially completed and empty CRFs) will be retained.

Follow-up of patients withdrawn from the study

In case of premature discontinuation after study drug intake, the investigations scheduled for the EOS visit will be performed 7±3 days after study drug discontinuation. The subjects will be advised that participation in these investigations is voluntary. Furthermore, they may request that from the time point of withdrawal no more data will be recorded and that all biological samples collected in the course of the study will be destroyed.

Replacement policy

All renal transplant recipients with HCV-infection, who are considered eligible for the participation in the present trial and who have given written informed consent, will be included in the present trial. Statistical analyses will include an intention-to-treat- and a per-protocol-set.

### Premature termination of the study

The sponsor has the right to close this study at any time. The IEC and the competent regulatory authority must be informed.

The trial or single dose steps will be terminated prematurely in the following cases:

- If adverse events occur which are so serious that the risk-benefit ratio is not acceptable.
- If the number of dropouts is so high that proper completion of the trial cannot realistically be expected.

# Methodology

## Screening investigation

All subjects, who are considered eligible for the participation in the present trial will undergo following procedures at the screening visit:

- Medical history

- Physical examination

- Vital signs (blood pressure, heart frequency), weight and height

- 12-lead ECG

## Dose selection, treatment duration and modification

CsA will be dosed as necessary to achieve 2h-CsA-serum concentrations of 500-1000 ng/dl.

## Study medication

Since only accredited preparations are being used and since the national health insurances are defraying for all medication costs; there are no special precautions to regard referring to medication management that are differing from standard storage in daily clinical routine. All medication is not blinded and used according to the Summary of Product characteristics.

Active agent and it´s characteristics: Cyclosporine A

Trade name of the agent: Sandimmun ®

Manufacturer: Novartis GmBH

Drug supply: Local pharmacy, i.e. external drugstore

Storage Instructions: Room temperature

Packing instructions: Cyclosporine A will obtained as Sandimmun ® in its trading package from local pharmacies, i.e. external drugstores.

Route of administration: per os

Referring to a reference point (product characteristics…)

### Dosage and administration

*Substance Cyclosporine A*

*Dose,forms and strenghts :* 10 mg, 25 mg, 50 mg and 100 mg capsules

initial dose: 10 mg/kg body weight/day

maintenance dose: dose adjustment according to the 2h-CsA serum level (goal: 500-1000 ng/ml)

*Route:* oral

*Administration:* per os

*Study-drug lock diary:* measurement of 2h-CsA serum concentrations

### Study-drug up- and down titration

Serum CsA levels will be measured 2 hours after the intake of CsA at each visit and if necessary for the achievement of target levels at additional timepoints. 2h-CsA goal levels will be 500-1000 ng/ml in all study participants independent of the time duration since renal transplantation to ensure CsA-serum concentrations previously shown to suppress HCV-replication *in vitro*.

### Study drug interruption or discontinuation

The investigator must temporarily interrupt or permanently discontinue the study drug if continued administration of the study drug is believed to be contrary to the best interests of the patient.

The interruption or premature discontinuation of study drug might be triggered by an AE, a diagnostic or therapeutic procedure, an abnormal assessment (e.g., laboratory abnormalities), or for administrative reasons, in particular withdrawal of the patient’s consent.

The reason for study drug interruption or premature permanent discontinuation must be documented in the CRF.

If the reason for premature permanent discontinuation of study treatment is an AE, the patient should have a “Premature EOS” visit with all the assessments performed before the study drug discontinuation, whenever possible.

If the reason for premature permanent discontinuation of study treatment is not an AE, the patient should be withdrawn from the study (withdrawal of consent) and have the EOS visit with all the assessments performed before the study drug discontinuation, whenever possible.

### Study-drug delivery & drug storage conditions

Since only accredited preparations are being used and since the national health insurances are defraying for all medication costs; there are no special precautions to regard referring to medication management that are differing from standard storage in daily clinical routine. Patients will be advised to store the drugs as recommended by the producer at room temperature.

### Study drug packaging and labeling

Study medication is used according to the summary of Product Characteristics. No special requirements for labeling are thus foreseen..

### IMP administration & handling

Administration and Handling will be done according to the SmPC.

### Drug Accountability

Drug Accountability will be recorded at on-going basis on paper form/source data and the date of drug dispensing by local, external pharmacies entered in the CRF at each visit. Furthermore the correct intake of IMP or any variations concerning that will be recorded in the CRF at each visit during treatment period.

Cyclosporine A will be prescribed as Sandimmun ® (trade name) in trading packages à 50 tablets, which will be obtained from local pharmacies, i.e. external drugstores, by the study participants themselves. All study participants will be asked to keep the used (empty) drug blisters and to return them to the study site for drug accountability. Drug accountability check will be performed at each visit.

### Concomitant medication

The well-being of the patient has the first priority, and modifications of concomitant treatment during the trial are allowed as necessary and will be documented in the patient’s records.

In this study, all concomitant medications are allowed, with the exception of:

Not allowed: HCV-specific antiviral therapy

### Interactions, reverse reactions and side effect of IMP:

*Cyclosporin A – interactions*

(according to http://medis.akhwien.at/cgi-bin/medisweb.exe?app_fach%20120689)

Caution when used with other nephrotoxic substances, e.g. aminoglycosides, amphotericin B, ciprofloxacin, melphalan, colchicine, trimethoprim, vancomycin, non-steroidal antiphlogistics, H2-receptor antagonists, methotrexate, lercarnidipine,...

Increase of cyclosporin A-serum concentrations by e.g., amphotericin B, keto-, flu-, itra- and voriconazole, erythro-, josa- and clarithromycin, doxycycline, oral contraceptives, propafenone, calcium-antagonists, metoclopramide, danazole, methylprednisolone, allopurinole, amiodarone, cholic acid, protease-inhibitors, imatinib, colchicine, nefazadone, ...

Decrease of cyclosporine A-serum concentrations by e.g., barbiturates, carbamazepine, oxcarbazepine, phenytoine, metamizole, rifampicin, nafcillin, sulfadimidine intravenous, trimethoprim, octreotide, probucol, orlistat, hypericum perforatum, ticlopidine, sulfinpyrazone, terbinafine, bosentan, ...

*Cyclosporin A – side effects*

(according to http://medis.akhwien.at/cgi-bin/medisweb.exe?app_fach%20120689)

| **system** | **frequency** |  |
| --- | --- | --- |
| *blood, lymph system* | >1/1000, <1/100 | anemia, thrombocytopenia |
|  | >1/10000, <1/1000 | leukopenia, microangiopathic hemolytic anemia, hemolytic-uremic syndrome |
|  | >1/1000, <1/100 | menstruation dysorders |
|  | >1/10000, <1/1000 | hyperthermia, gynaecomastia, hot flushes |
| *metabolism* | >1/10 | hyperlipidemia |
|  | >1/100, <1/10 | anorexia, hyperuricemia |
|  | >1/1000, <1/100 | hyperkalemia, hypomagnesemia, weight gain, hyperglycemia |
| *nervous system* | >1/10 | tremor, head ache |
|  | >1/100, <1/10 | fatigue, parasthesia |
|  | >1/1000, <1/100 | signs of encephalopathy (e.g. convulsions, desorientation, sleeping disorders) |
|  | >1/10000, <1/1000 | motoric polyneuropathy |
|  | <1/1000 | deafness, papillary edema |
| *cardiovascular* | >1/10 | hypertension |
|  | >1/100, <1/10 | ischemic heart disease |
| *gastrointestinal* | >1/100, <1/10 | gingivitis hypertrophicans, gastrointestinal symptoms |
|  | >1/1000, <1/100 | gastric ulcera |
|  | >1/10000, <1/1000 | pancreatitis |
|  | <1/1000 | colitis |
| *liver, bile* | >1/1000, <1/100 | reversible increase of bilirubin and parameters of liver function |
| *skin* | >1/100, <1/10 | hypertrichosis |
|  | >1/1000, <1/100 | acne, rash |
|  | >1/10000, <1/1000 | redness, pruritus |
| *musculo-skeletal* | >1/100, <1/10 | seizures, muscle pain |
|  | >1/10000, <1/1000 | myopathy, weakness |
| *kidney, urinary tract* | >1/10 | increase of serum creatinine |

### Randomization and stratification:

Not applicable

### Blinding

Not applicable

**7.3.12. Benefit and risk assessment**

Benefits

- Reduction of HCV-replication and improvement of liver function with consecutive improvement of quality of life and reduced HCV-associated morbidity and mortality

Risks:

- Potential increase of serum creatinine after conversion, which however is anticipated to be reversible. In case of a significant decrease of graft function, defined as 50% reduction of the glomerular filtration rate (GFR) in subjects with a GFR ≥50 ml/min at baseline and as 30% reduction of the GFR in subjects with a GFR <50 ml/min at baseline, the study drug will be discontinued in the respective study participant and immunosuppression re-converted to a tacrolimus-based regimen.

In case of serious side effects or serious other medical safety concerns, single study participants will be withdrawn or the whole study prematurely terminated; the immunosuppression will be re-converted to a tacrolimus-based regimen.

## Study procedures

### Description of study procedures

*7.4.1.1. Switch of immunosuppression*

Serum CsA levels will be measured 2 hours after the intake of CsA at each visit and if necessary for the achievement of target levels at additional timepoints. 2h-CsA goal levels will be 500-1000 ng/ml. In addition, parameters of renal function including serum creatinine and protein excretion in 24h-urine will be determined every four weeks during the study period according to local standards of routine care.

*7.4.1.2. Evaluation of liver function*

*7.4.1.2.1. Transient elastography*

Parameters of liver stiffness will be assessed by transient elastography (FibroScan®, Echosens, Paris France), as described previously [49]. Transient elastography represents a novel approach for staging of hepatic fibrosis and has been tested in multiple study collectives with varying degrees of liver fibrosis and cirrhosis including subjects with hepatitis C. This technique rapidly and non-invasively measures the mean hepatic stiffness by means of an ultrasonic transducer, which transmits a vibration of low frequency (50 Mhz) and amplitude into the liver. The vibration wave induces an elastic shear wave that propagates through the liver tissue. The velocity of this wave as it passes through the liver correlates directly with tissue stiffness. The harder or stiffer the tissue, the faster the shear wave propagates.

All measurements will be performed on the right lobe of the liver through intercostal spaces on patients lying in the dorsal decubitus position with the right arm in maximal abduction. The tip of the transducer probe will be placed in an intercostal space at the level of the right lobe of the liver. A liver portion of 6 cm thickness free of large vascular structures will be located before the measurement will be started by pressing the probe button. The results will be expressed in kilopascal (kPa) and the median value considered representative of the liver stiffness. Only procedures with 10 valid measurements, a success rate (= ratio of the number of successful measurements over the total number of acquisitions) ≥ 80% and an interquartile range (IQR) < 10 kPa will be considered reliable. Time expenditure for the assessment of liver stiffness by transient elastography is estimated at 5 minutes per patient.

The advantages of transient elastography include non-invasiveness, reproducibility, quick and painless examination as well as reduced sampling error (due to the large liver volume of 6x1 cm as compared to the size of a standard liver biopsy).

*7.4.1.2.2. Biochemical evaluation*

HCV genotypes will be identified by Line Probe assay (Innogenetics NV, Zwijnaarde, Belgium). HCV virusload will be determined by Cobas Amplicor HCV Monitor v2.0 (Roche Diagnostics Systems, Branchburg, NJ, USA) to evaluate viral kinetics if the virusload is over the lower limit of detection. To evaluate virus negativity qualitative HCV tests will be performed using the Cobas Amplicor HCV (Roche Diagnostics Systems, Branchburg, NJ, USA). IP-10 will be measured as described previously [17].

*7.4.1.3. Oral Glucose Tolerance Test (OGTT)*

2h‑75g-OGTTs will be performed in the morning (8 a.m.) after an overnight fast, respectively. Prior to and 30, 60, 90 and 120 min after the ingestion of the glucose solution (Gluco‑Drink 75®, Roche Diagnostics GmbH, Vienna, Austria), blood samples will be obtained for the determination of glucose, insulin, C‑peptide and free fatty acid (7x9 ml blood). In addition, blood samples for the measurement of circulating adipokine levels will be taken at time-points 0, 30, 60 and 120 min (4x9ml blood). In total, 99-104 ml blood will be drawn.

*Evaluation of Insulin Sensitivity, Insulin Secretion and Overall Glucose Metabolism*: To assess changes from baseline, areas under the curve of plasma glucose, insulin, C-peptide and FFAs during the OGTT will be computed by trapezoidal integration. Insulin sensitivity during the OGTT will be assessed by the oral glucose insulin sensitivity (OGIS) index, which quantifies dynamic glucose clearance per unit change of insulin [50]. OGIS has been validated against insulin sensitivity data derived from gold-standard euglycaemic-hyperinsulinemic clamps [50, 51] and has been previously used in various metabolic studies [52-54]. Basal insulin sensitivity will be evaluated by the homeostasis model assessment of insulin resistance (HOMA-IR) index and calculated as fasting insulin concentration (µU/ml) * fasting glucose concentration (mmol/l) /22.5.

In addition, OGTT concentration data of glucose, C‑peptide and insulin will be analysed by means of mathematical models describing their respective kinetics. These models provide a good estimation of total B‑cell insulin secretion rate (TIS), hepatic insulin extraction (HIE) [55, 56] as well as of adaptation and disposition index. Based on the measurement of C‑peptide, the adaptation index yields an estimation of the pre‑hepatic insulin‑secretory capacity of pancreatic beta‑cells in response to changes of the prevailing insulin sensitivity and, thus, provides a measurement of beta-cell insulin secretion unaffected by HIE [57-59]. The disposition index relates the post‑hepatic insulin response to changes in insulin sensitivity [60] and represent a marker of overall glucose metabolism considering both insulin secretion and HIE in relation to the prevailing insulin sensitivity.

*7.4.1.4. Biochemical measurements*

Plasma samples will be frozen at -80°C until subsequent analysis.

General rules for trial procedures

- All study measures like blood sampling and measurements (vital parameters, ECG, etc.) have to be documented with date (dd:mm:yyyy).
- In case several study procedures are scheduled at the same time point, there is no specific sequence that should be followed.
- The dates of all procedures should be according to the protocol. The time margins mentioned in the study flow chart are admissible. If for any reason, a study procedure is not performed within scheduled margins a protocol deviation should be noted, and the procedure should be performed as soon as possible or as adequate.
- If it is necessary for organizational reasons, it is admissible to perform procedures which are scheduled for one visit at two different time points. Allowed time margins should thereby not be exceeded.

7.4.1.1. Screening and screening procedure

All study participants will be recruited among the patients with prior renal transplantation and known HCV-infection, who attend the Department of Internal Medicine III, Division of Nephrology and Dialysis, Medical University of Vienna, for standard care.

All subjects, who are considered eligible for the participation in the present trial will undergo following procedures at the screening visit (= visit 1):

- Medical history

- Physical examination

- Vital signs (blood pressure, heart frequency), weight and height

- 12-lead ECG

7.4.1.2. Randomization:

Not applicable

**7.4.1.3. Treatment**

In the present trial, immunsuppression will be converted from a tacrolimus- to a CsA-based regimen in all study participants. The total CsA-treatment period is 12 weeks.

7.4.1.4. End-of-study (EOS) examination

After a treatment period of 12 weeks patients undergo the end-of-study examination that entails following procedures:

- Biochemical measurements including potassium, sodium, chloride, calcium, phosphate, magnesium, creatinine, urea, uric acid, triglycerides, total cholesterol, HDL-C, LDL-C, total bilirubin, total alkaline phosphatase, ALT, AST, GGT, cholinesterase, LDH, CK, albumin, CRP, blood cell count, clotting tests, oral glucose tolerance test, HbA1c, glycated albumin, plasma adipokine levels, 24-urine, TSH, 25OH-vitamin D, 1,25OH-vitamin D, alpha-fetoprotein, IP-10, cryoglobulins, rheuma factors, serum 2h-CsA or FK506-levels (as applicable) and HCV-RNA

- Oral glucose tolerance test

- Transient elastography

- Physical examination

In case of a premature discontinuation, e.g. withdrawal of the informed consent or pregnancy (= Premature EoS), subjects will undergo the following procedures:

- Physical examination

- Vital parameters (blood pressure, heart frequency), weight and height

- Biochemical measurements including potassium, sodium, chloride, calcium, phosphate, magnesium, creatinine, urea, uric acid, triglycerides, total cholesterol, HDL-C, LDL-C, total bilirubin, total alkaline phosphatase, ALT, AST, GGT, cholinesterase, LDH, CK, albumin, CRP, blood cell count, clotting tests

# SAFETY DEFINITIONS AND REPORTING REQUIREMENTS

## 8.1 Adverse events (AEs)

### 8. 1. 1 Summary of known and potential risks of the study drug

*Cyclosporin A – side effects*

(according to http://medis.akhwien.at/cgi-bin/medisweb.exe?app_fach%20120689)

| **system** | **frequency** |  |
| --- | --- | --- |
| *blood, lymph system* | >1/1000, <1/100 | anemia, thrombocytopenia |
|  | >1/10000, <1/1000 | leukopenia, microangiopathic hemolytic anemia, hemolytic-uremic syndrome |
|  | >1/1000, <1/100 | menstruation dysorders |
|  | >1/10000, <1/1000 | hyperthermia, gynaecomastia, hot flushes |
| *metabolism* | >1/10 | hyperlipidemia |
|  | >1/100, <1/10 | anorexia, hyperuricemia |
|  | >1/1000, <1/100 | hyperkalemia, hypomagnesemia, weight gain, hyperglycemia |
| *nervous system* | >1/10 | tremor, head ache |
|  | >1/100, <1/10 | fatigue, parasthesia |
|  | >1/1000, <1/100 | signs of encephalopathy (e.g. convulsions, desorientation, sleeping disorders) |
|  | >1/10000, <1/1000 | motoric polyneuropathy |
|  | <1/1000 | deafness, papillary edema |
| *cardiovascular* | >1/10 | hypertension |
|  | >1/100, <1/10 | ischemic heart disease |
| *gastrointestinal* | >1/100, <1/10 | gingivitis hypertrophicans, gastrointestinal symptoms |
|  | >1/1000, <1/100 | gastric ulcera |
|  | >1/10000, <1/1000 | pancreatitis |
|  | <1/1000 | colitis |
| *liver, bile* | >1/1000, <1/100 | reversible increase of bilirubin and parameters of liver function |
| *skin* | >1/100, <1/10 | hypertrichosis |
|  | >1/1000, <1/100 | acne, rash |
|  | >1/10000, <1/1000 | redness, pruritus |
| *musculo-skeletal* | >1/100, <1/10 | seizures, muscle pain |
|  | >1/10000, <1/1000 | myopathy, weakness |
| *kidney, urinary tract* | >1/10 | increase of serum creatinine |

### 8. 1.2 Definition of adverse events

An AE is any adverse change from the subject's baseline condition, i.e., any unfavorable and unintended sign including an abnormal laboratory finding, symptom or disease which is considered to be clinically relevant be the physician that occurs during the course of the study, whether or not considered related to the study drug.

Adverse events include:

- Exacerbation of a pre-existing disease.
- Increase in frequency or intensity of a pre-existing episodic disease or medical condition.
- Disease or medical condition detected or diagnosed after study drug administration even though it may have been present prior to the start of the study.
- Continuous persistent disease or symptoms present at baseline that worsen following the start of the study.
- Lack of efficacy in the acute treatment of a life-threatening disease.
- Events considered by the investigator to be related to study-mandated procedures.
- Abnormal assessments, e.g., ECG and physical examination findings, must be reported as AEs if they represent a clinically significant finding that was not present at baseline or worsened during the course of the study.
- Laboratory test abnormalities must be reported as AEs if they represent a clinically significant finding, symptomatic or not, which was not present at baseline or worsened during the course of the study or led to dose reduction, interruption or permanent discontinuation of study drug.

Adverse events do not include:

- Medical or surgical procedure, e.g., surgery, endoscopy, tooth extraction, transfusion. However, the event leading to the procedure is an AE. If this event is serious, the procedure must be described in the SAE narrative.
- Pre-existing disease or medical condition that does not worsen.
- Situations in which an adverse change did not occur, e.g., hospitalizations for cosmetic elective surgery or for social and/or convenience reasons.
- Overdose of either study drug or concomitant medication without any signs or symptoms. However, overdose must be mentioned in the Study Drug Log.

## 8. 2 Serious adverse events (SAEs)

A Serious Adverse Event (SAE) is defined by the International Conference on Harmonization (ICH) guidelines as any AE fulfilling at least one of the following criteria:

- results in deaths.
- Life-threatening – defined as an event in which the subject was, in the judgment of the investigator, at risk of death at the time of the event; it does not refer to an event that hypothetically might have caused death had it been more severe.
- Requiring subject's hospitalization or prolongation of existing hospitalization – inpatient hospitalization refers to any inpatient admission, regardless of length of stay.
- Resulting in persistent or significant disability or incapacity (i.e., a substantial disruption of a person’s ability to conduct normal life functions).
- Congenital anomaly or birth defect.
- Is medically significant or requires intervention to prevent at least one of the outcomes listed above.

Life-threatening refers to an event in which the subject was at risk of death at the time of the event. It does not refer to an event that hypothetically might have caused death if it were more severe.

Important medical events that may not immediately result in death, be life-threatening, or require hospitalization may be considered as SAEs when, based upon appropriate medical judgment, they may jeopardize the subject and may require medical or surgical intervention to prevent one of the outcomes listed in the definitions above.

### 8. 2. 1 Hospitalization – Prolongation of existing hospitalization

Hospitalization is defined as an overnight stay in a hospital unit and/or emergency room.

An additional overnight stay defines a prolongation of existing hospitalization.

The following is not considered an SAE and should be reported as an AE only:

- Treatment on an emergency or outsubject basis for an event not fulfilling the definition of seriousness given above and not resulting in hospitalization.

The following reasons kfor hospitalizations are not considered AEs, and therefore not SAEs:

- Hospitalizations for cosmetic elective surgery, social and/or convenience reasons.
- Standard monitoring of a pre-existing disease or medical condition that did not worsen, e.g., hospitalization for coronary angiography in a subject with stable angina pectoris.
- Elective treatment of a pre-existing disease or medical condition that did not worsen, e.g., hospitalization for chemotherapy for cancer, elective hip replacement for arthritis.

**8.2.2** SAEs related to study-mandated procedures

Such SAEs are defined as SAEs that appear to have a reasonable possibility of causal relationship (i.e., a relationship cannot be ruled out) to study-mandated procedures (excluding administration of study drug) such as discontinuation of subject's previous treatment during a washout period, or complication of a mandated invasive procedure (e.g., blood sampling, heart catheterization), or car accident on the way to the hospital for a study visit, etc.

### 8. 2. 3 Suspected unexpected serious adverse reactions (SUSARs)

SUSARs are all suspected adverse reactions related to the study drug that are both unexpected (not previously described in the SmPC - Summary of Product Characteristics or Investigator’s brochure) and serious.

**8.2.4. Pregnancy**

Any pregnancy that occurs during study participation must be reported to the principal investigator/sponsor. **To ensure subject safety, each pregnancy must be reported to the principal investigator/sponsor immediately** The pregnancy must be followed up to determine outcome (including premature termination) and status of mother and child. Pregnancy complications and elective terminations for medical reasons must be reported as an AE or SAE. Spontaneous abortions must be reported as an SAE.

Any SAE occurring in association with a pregnancy, brought to the investigator’s attention after the subject has completed the study and considered by the investigator as possibly related to the investigational product, must be promptly reported to the principal investigator/sponsor.

In addition, the investigator must attempt to collect pregnancy information on any female partners of male study subjects who become pregnant while the subject is enrolled in the study. Pregnancy information must be reported to the principal investigator/sponsor as described above.

Address of Sponsor:

Univ.-Prof. DDr. Walter H. Hörl, Medical University of Vienna, Department of Internal Medicine III, Division of Nephrology and Dialysis, Waehringer Guertel 18-20, 1090 Vienna, Austria

Address of Investigator:

Univ.-Prof. Dr. Alice Schmidt, Medical University of Vienna, Department of Internal Medicine III, Division of Nephrology and Dialysis, Waehringer Guertel 18-20, 1090 Vienna, Austria

## 8. 3 Severity of adverse events

The severity of clinical AEs is graded on a three-point scale: mild, moderate, severe, and reported on specific AE pages of the CRF.

If the severity of an AE worsens during study drug administration, only the worst intensity should be reported on the AE page. If the AE lessens in intensity, no change in the severity is required.

If an AE occurs during a washout or placebo run-in phase and afterwards worsens during the treatment phase, a new AE page must be filled in with the intensity observed during study drug administration.

- **Mild**

Event may be noticeable to subject; does not influence daily activities; the AE resolves spontaneously or may require minimal therapeutic intervention;

- **Moderate**

Event may make subject uncomfortable; performance of daily activities may be influenced; intervention may be needed; the AE produces no sequelae.

- **Severe**

Event may cause noticeable discomfort; usually interferes with daily activities; subject may not be able to continue in the study; the AE produces sequelae, which require prolonged therapeutic intervention.

A mild, moderate or severe AE may or may not be serious. These terms are used to describe the intensity of a specific event (as in mild, moderate, or severe myocardial infarction). However, a severe event may be of relatively minor medical significance (such as severe headache) and is not necessarily serious. For example, nausea lasting several hours may be rated as severe, but may not be clinically serious. Fever of 39°C that is not considered severe may become serious if it prolongs hospital discharge by a day. Seriousness rather than severity serves as a guide for defining regulatory reporting obligations.

**8. 4** Relationship to study drug

For all AEs, the investigator will assess the causal relationship between the study drug and the AE using his/her clinical expertise and judgment according to the following algorithm that best fits the circumstances of the AE:

- Unrelated
  - May or may not follow a reasonable temporal sequence from administration of the study product
  - Is biologically implausible and does not follow known response pattern to the suspect study drug (if response pattern is previously known)
  - Can be explained by the known characteristics of the subject’s clinical state or other modes of therapy administered to the subject.
- Possible related
  - Follows a reasonable temporal sequence form administration of the study drug.
  - May follow a known response pattern to the study drug (if response pattern is previously known).
  - Could not be reasonable not be reasonably explained by the known characteristics of the subject’s clinical state or other modes of therapy administered to the subject, if applicable.
- Definitely related
  - Follows a reasonable temporal sequence form administration of the study drug.
  - Follows a known response pattern to the study drug (if response pattern is previously known).
  - No other reasonable cause is present.

## 8. 5 Reporting procedures

A special section is designated to adverse events in the case report form. The following details must thereby be entered:

- Type of adverse event
- Start (date and time)
- End (date and time)
- Severity (mild, moderate, severe)
- Serious (no / yes)
- Unexpected (no / yes)
- Outcome (resolved, ongoing, ongoing – improved, ongoing – worsening)
- Relation to study drug (unrelated, possibly related, definitely related)

Adverse events are to be documented in the case report form in accordance with the above mentioned criteria.

### 8. 5. 1 Reporting procedures for SAEs

In the event of serious, the investigator has to use all supportive measures for best patient treatment. A written report is also to be prepared and made available to the clinical investigator immediately.. The following details should at least be available:

- Patient initials and number
- Patient: date of birth, sex, ethical origin
- The suspected investigational medical product (IMP)
- The adverse event assessed as serious
- Short description of the event and outcome
- If applicable, the initial report should be followed by the Follow up report, indicating the outcome of the SAE.

### 8. 5. 2 Reporting procedures for SUSARs

It must be remembered that the regulatory authorities, and in case of SUSARs which could possibly concern the safety of the study participants, also the Institutional Review Board / Independent Ethics Committee (IRB / IEC) are to be informed. Such reports shall be made by the study management and the following details should be at least available:

- Patient initials and number
- Patient: date of birth, sex, ethical origin
- Name of investigator and investigating site
- Period of administration
- The suspected investigational medical product (IMP)
- The adverse event assessed as serious and unexpected, and for which there is a reasonable suspected causal relationship to the IMP
- Concomitant disease and medication
- Short description of the event:
  - Description
  - Onset and if applicable, end
  - Therapeutic intervention
  - Causal relationship
  - Hospitalization of prolongation of hospitalization
  - Death, life-threatening, persistent or significant disability or incapacity

Electronic reporting should be the expected method for reporting of SUSARs to the competent authority. In that case, the format and content as defined by Guidance (28) should be adhered to. The latest version of MedDRA should be applied. Lower level terms (LLT) should be used.

### 8. 5. 3 Annual Safety Report

The Annual Safety Report will be provided by the principal investigator at least once a year.

This report will also be presented annually to the Institutional Review Board / Independent Ethics Committee (IRB / IEC) and to the competent authorities by the sponsor.

# Follow-up

## Follow-up of study participants including follow-up of adverse events

In the present study, study participants will be recruited among the patients who attend the Department of Internal Medicine III, Division of Nephrology and Dialysis, of the Medical University of Vienna for standard care after renal transplantation. All patients undergo regular visits at certain time-intervals, which depend on the time since the transplantation or on the evaluation of the physician (short-term control visit in case of an adverse event, e.g. increase of serum creatinine, infection, or change of immunosuppression). Additionally, patients have the possibility to attend our site in case of an adverse event, e.g. fever, urinary tract infection, diarrhoea, without appointment.

After the end of the study, all study participants will be taken care of in this routine care setting similar to before participation in the study.

## Treatment after end of study

The immunosuppressive regimen after the end of study will depend on the response of HCV-replication on CsA-treatment. Subjects, who have shown a decrease of HCV-RNA of at least log -2, will continue with a CsA-based immunosuppression. Subjects, who did not respond with an attenuation of HCV-replication will be treated with their original tacrolimus-based immunosuppressive treatment-scheme.

# STATISTICAL METHODOLOGY AND ANALYSIS

## Analysis sets

Two different analysis sets are defined:

- **Intention to treat set**

This analysis set includes subjects who received at least one dose study drug,

- **Per-protocol set**

This analysis set comprises all subjects who received study drug (at least one dose) and did not violate the protocol in a way that might affect the evaluation of the effect of the study drug(s) on the primary objective, i.e., without major protocol violations.

## Sample size considerations

A sample size of 26 will have 90% power to detect a difference in means of 1, assuming a standard deviation of differences of 1,5, using a paired t-test with a 0,050 two-sided significance level. Considering a drop-out rate of 10 percent results in a sample size of about 30 patients. If more patients are willing to participate in the study, up to 50 patients will be recruited. In this case more refined mean differences can be detected or a possible higher standard deviation of differences can be accounted for.

Last values will be carried forward (LVCF) in the case of missing values in the outcome variables for the intention to treat analyses. An investigation will be made concerning the sensitivity of the results of analysis to the method of handling missing values, especially if the number of missing values is substantial.

## Relevant protocol deviations

All protocol deviations will be listed in the study report.

## 10. 4 Endpoints analysis

### 10. 4. 1 Primary endpoint analysis

The primary endpoint HCV-RNA will be logarithmically transformed (base 10). The paired t-test used to assess the effect of a conversion from a tacrolimus- to a cyclosporine A-based immunosuppression on HCV-replication after 12 weeks.

### 10. 4. 2 Secondary endpoint analysis

Secondary endpoints will be analysed with descriptive and exploratory statistical standard tools.

### 10. 4. 3 Safety and tolerability endpoints

Safety and tolerability endpoints will be reported by use of descriptive statistics. Their relevance will be assessed from a clinical perspective.

### 10. 4. 4 Baseline parameters and concomitant medications

Baseline parameters and concomitant medications will be reported by use of descriptive statistics.

##

## 10. 5 Exploratory analyses

Secondary endpoints will be analysed in an exploratory manner. No adjustments for multiple testing will be performed.

##

## 10. 6 Interim analysis

No interim analysis is planned.

##

## 10. 7 Software program(s)

NQUERY (Statistical Solutions Ltd., Cork, Ireland) has been used for study planning. Microsoft Excel, SAS (SAS Institute Inc., Cary, NC) and SPSS (SPSS Inc., Chicago, IL) will be used for statistical analyses.

# 11 DOCUMENTATION AND DATA MANAGEMENT

## 11. 1 Documentation of study results

A subject screening and enrollment Log will be completed for all eligible or non-eligible subjects with the reasons for exclusion.

### 11. 1. 1 Case report form (CRF)

For each subject enrolled, regardless of study drug initiation, a “Paper-CRF” must be completed and signed by the principal investigator or co-investigator. This also applies to those subjects who fail to complete the study. If a subject withdraws from the study, the reason must be noted on the CRF. Case report forms are to be completed on an ongoing basis.

CRF entries and corrections will only be performed by study site staff, authorized by the investigator.

In the “Paper-CRF” all forms will be completed using a black pen and must be legible*. Errors should be crossed out but not obliterated, the correction inserted, and the change initialed and dated by the investigator, co-investigator or study nurse.

The entries will be checked by trained personnel (Monitor) and any errors or inconsistencies will be checked immediately.

The original CRFs will be passed.to the data manager Dr. Ammon Handisurya, Department of Internal Medicine III, Division of Nephrology and Dialysis, Medical University of Vienna.

**11.1.2 Data Collection**

Data collected at all visits are entered into an interactive form. The CRFs will be source documents verified following guidelines established before study onset as detailed in the Monitoring Plan. Maintenance of the study database will be performed by Dr. Ammon Handisurya, Department of Internal Medicine III, Division of Nephrology and Dialysis, Medical University of Vienna.

## 11. 2 Safekeeping

The investigator will maintain adequate and accurate records to enable the conduct of the study to be fully documented and the study data to be subsequently verified. These documents will be classified into two different categories: investigator's file, and subject clinical source documents.

The investigator's file will contain the protocol/amendments, EudraCT forms, CRFs (eCRF printout), standard operation procedures (SOPs), data clarification and query forms, EC/IRB and Health Authority approval with correspondence, informed consent, drug records, staff curriculum vitae and authorization forms, screening and enrollment logs, and other appropriate documents/correspondence as per ICH/Good Clinical Practice (GCP) and local regulations.

Subject clinical source documents include, but are not limited to subject hospital/clinic records, physician’s and nurse’s notes, appointment book, original laboratory reports, ECG, X-ray, pathology and special assessment reports, consultant letters, etc.

These two categories of documents must be kept on file by the investigator for as long as needed to comply with national and international regulations (in Austria 15 years after discontinuing clinical development or after the last marketing approval). If source documents are not durable as long as needed (e.g. ECG printouts) they must be preserved as a copy. No study document should be destroyed without prior written approval from the Department of Internal Medicine III, Division of Nephrology and Dialysis, Medical University of Vienna.

When source documents are required for the continued care of the subject, appropriate copies should be made for storing outside of the site.

## 11. 3 Monitoring

The monitor will contact and visit the investigator regularly and will be allowed, on request, to have access to all source documents needed to verify the entries in the CRFs and other protocol-related documents provided that subject confidentiality is maintained in agreement with local regulations. It will be the monitor's responsibility to inspect the CRFs at regular intervals throughout the study, to verify the adherence to the protocol and the completeness, consistency and accuracy of the data being entered on them. The monitoring standards require full verification for the presence of informed consent, adherence to the inclusion/exclusion criteria, documentation of SAEs and the recording of the main efficacy, safety, and tolerability endpoints.

To be GCP compliant at least 3 monitoring visits are scheduled. An initiation visit, one routine visit and a final visit after the last patient has finished the study. The monitor will be working according to SOPs and will provide a GCP-compliant monitoring report after each visit for the sponsor and the investigator. Depending on the quality of the data, additional monitoring visits will be necessary according to the sponsor’s discretion.

Monitoring will be performed by Univ.-Prof.Dr. Anna-Christine Hauser, Department of Internal Medicine III, Division of Nephrology and Dialysis, Medical University of Vienna, Austria.

**11.4 Quality Control and Quality Assurance**

**11.4.1 Periodic Monitoring**

A designated CRA will inspect the paper CRF at regular intervals throughout the study to verify completeness, accuracy and consistency of the data and adherence to the protocol and GCP guidelines. The CRA should have to access to all source data needed to verify the entries in the paper CRFs. The investigator will cooperate with the CRA to ensure that any identified discrepancies are resolved.

Monitoring visits will be performed three times during the study period (an initiation visit, one routine visit and a final visit after the last patient has finished the study) and 100% source data will be checked by the monitor (100% “Source Data Verification”, SDV)

**11.4.2 Audit and Inspections**

Upon request, the investigator will make all study-related source data and records available to a qualified quality assurance auditor mandated by the sponsor or to CA inspectors. The main purpose of an audit or inspection are to confirm that the rights and welfare of the subjects have been adequately protected, and that all data relevant for assessment of safety and efficacy of the investigational product have appropriately been reported to the sponsor.

## 11.5 Reporting and Publication

**11.5.1 Clinical Study Report (CSR)**

Within one year after the final completion of the study, a full CSR will be written by Univ.-Prof. Dr. Alice Schmidt and Dr. Ammon Handisurya, Department of Internal Medicine III, Division of Nephrology and Dialysis, Medical University of Vienna, and submitted to the EC and the competent authority.

The Principal Investigator will be asked to review and sign the final study report.

**11.5.2 Publication of study results**

The findings of this study will be published by the investigators in a scientific journal and presented at scientific meetings. The manuscript will be circulated to all co-investigators before submission.

# ETHICAL AND LEGAL ASPECTS

## 12. 1 Informed consent of subjects

Following comprehensive instruction regarding the nature, significance, impact and risks of this clinical trial, the patient must give written consent to participation in the study.

During the instruction the patients are to be made aware of the fact that they can with-draw their consent – without giving reasons – at any time without their further medical care being influenced in any way.

In addition to the comprehensive instructions given to the patients by the investigator, the patients also receive a written patient information sheet in comprehensible language, explaining the nature and purpose of the study and its progress.

The patients must agree to the possibility of study-related data being passed on to relevant authorities.

The patients must be informed in detail of their obligations in relation to the proband insurance in order not to jeopardize insurance cover.

## 12. 2 Acknowledgement / approval of the study

The investigator will submit this protocol and any related document provided to the subject (such as subject information used to obtain informed consent) to an Ethics Committee (EC) or Institutional Review Board (IRB). Approval from the committee must be obtained before starting the study, and should be documented in a dated letter to the investigator.

Adverse events - whether serious and/or unexpected, and possibly endangering the safety of the study participants - are likewise to be reported to the ethics committee.

The clinical trial shall be performed in full compliance with the valid legal regulations according to the Drug Law (AMG - Arzneimittelgesetz) of the Republic of Austria.

The study must be notified to the Austrian Agency for Health and Food Safety (AGES) and to the European Agency for the Evaluation of Medicinal Products (EMEA) and registered to the European Clinical Trial Database (EudraCT) using the required forms.

### 12. 2 .1 Changes in the Conduct of the Study

### 12.2.1.1 Protocol amendments

Proposed amendments must be submitted to the appropriate CA and ECs. Substantial amendments may be implemented only after CA/EC approval has been obtained. Amendments that are intended to eliminate an apparent immediate hazard to subjects may be implemented prior to receiving CA/EC approval. However, in this case, approval must be obtained as soon as possible after implementation.

### 12.2.1.2 Study Termination

If the sponsor or the investigator decides to terminate the study before it is completed, they will notify each other in writing stating the reasons of early termination. In terminating the study, the sponsor and the investigator will ensure the adequate consideration is given to the protection of the subject interests. The investigator, sponsor or designated CRO will notify the relevant CA and EC. Documentation will be filed in the Trial Master and Investigator Files.

## 12. 3 Finance and insurance

During their participation in the clinical trial the patients will be insured as defined by legal requirements. The principal investigator of the clinical trial will receive a copy of the insurance conditions of the 'patients insurance'. The sponsor is providing insurance in order to indemnify (legal and financial coverage) the investigator/center against claims arising from the study, except for claims that arise from malpractice and/or negligence. The compensation of the subject in the event of study-related injuries will comply with the applicable regulations.

Details on the existing patients insurance are given in the patient information sheet.

## 12. 4 Confidentiality

The information contained in this document, especially unpublished data, is the property of the Medical University of Vienna, Department of Internal Medicine III, Division of Nephrology and Dialysis It is therefore provided to you in confidence as an investigator, potential investigator, or consultant, for review by you, your staff, and an Ethics Committee or Institutional Review Board. It is understood that this information will not be disclosed to others without written authorization from the Principal Investigator except to the extent necessary to obtain informed consent from those persons to whom the study drug may be administered.

## 12. 5 Ethics and Good Clinical Practice (GCP)

The investigator will ensure that this study is conducted in full conformance with the principles of the "Declaration of Helsinki" (as amended at the 56^th^ WMA General Assembly, Tokyo, Japan, 2004) and with the laws and regulations of the country in which the clinical research is conducted.

The principal investigator of the clinical trial shall guarantee that only appropriately trained personnel will be involved in the study. All studies must follow the ICH GCP Guidelines (June 1996) and, if applicable, the Code of Federal Regulations (USA). In other countries in which GCP Guidelines exist, the investigators will strictly ensure adherence to the stated provisions.

Therefore this study follows the ICH GCP Guidelines embedded in the Austrian drug act.

# 13 APPENDICES

- Informed Consent Form (Version 1.0; Date 01-FEB-2011 )
- CRF (Version 1.0; Date 01-FEB-2011)

# REFERENCES

1. Alter MJ: Epidemiology of hepatitis C virus infection. World J Gastroenterol 2007; 13: 2436-2441
2. Poynard T, Bedossa P, Opolon P. Natural history of liver fibrosis progression in patients with chronic hepatitis C. The OBSVIRC, METAVIR, CLINIVIR, and DOSVIRC study groups. Lancet 1997; 349:; 825-832.
3. Pereira BJ, Wright TL, Schmid CH et al. for the New England Organ Bank Hepatitis C Study Group. The impact of pretransplantation hepatitis C infection on the outcome of renal transplantation. Transplantation 1995; 60: 799-805
4. Toth CM, Pascual M, Chung RT et al. Hepatitis C virus-associated fibrosing cholestatic hepatitis after renal transplantation. Transplantation 1998; 66: 1264-1258
5. Fabrizi F, Martin P, Dixit V et al. Hepatitis C virus antibody status and survival after renal transplantation: meta-analysis of observational studies. Am J Transplant 2005; 5: 1452-1461
6. Roth D, Cirocco R, Zucker K et al. De novo membranoproliferative glomerulonephritis in hepatitis C virus-infected renal allograft recipients. Transplantation 1995; 59: 1676-1682
7. Morales JM, Pascual-Capdevila J, Campistol JM et al. Membranous glomerulonephritis associated with hepatitis C virus infection in renal transplant recipients. Transplantation 1997; 63: 1634-1639
8. Baid S, Pascual M, Williams WW et al. Renal thrombotic microangiopathy associated with cardiolipin antibodies in hepatitis C-positive renal transplant recipients. J Am Soc Nephrol 1999; 10: 146-153
9. Fabrizi F, Martin P, Dixit V, et al. Post-transplant diabetes mellitus and HCV seropositive status after renal transplantation: a meta-analysis of clinical studies. Am J Transplant. 2005; 5: 2433-2440
10. Ghany MG, Strader DB, Thomas DL et al. American Association for the Study of Liver Diseases: Diagnosis, management, and treatment of hepatitis C: An update. Hepatology 2009; 49: 1335-1374
11. Black M, Peters M. Alpha-interferon treatment of chronic hepatitis C: Need for accurate diagnosis in selscting patients. Ann Intern Med 1992: 116: 86-88
12. Martin P, Fabrizi F: Treatment of chronic hepatitis C infection in patients with renal failure. Clin Gastroenterl Hepatol 2005; 3 (10 Suppl 3): S113-7
13. Genetic testing for cystic fibrosis. NIH Consensus Statement 1997; 15: 1-37
14. Abboud O, Becker G, Bellorin-Font E et al. KDIGO Clinical Practive Guidelines for the prevention, diagnosis, evaluation, and treatment of hepatitis C in chronic kidney disease. Kidney Int Suppl 2008; 109: S1-99
15. Kamar N, Sandres-Saune K, Selves J et al. Long-term ribavirin therapy in hepatitis C virus-positive renal transplant patients: Effects on renal function and liver histology. Am J Kidney Dis 2003; 42: 184-192
16. Fontaine H, Vallet-Pichard A, Equi-Andrade C et al. Histopathologic efficacy of ribavirin monotherapy in kidney allograft recipients with chronic hepatitis C. Transplantation 2004; 78: 853-857
17. Reiberger T, Aberle JH, Kundi M et al: IP-10 correlates with hepatitis C viral load, hepatic inflammation and fibrosis and predicts hepatitis C virus relapse and non-response in HIV-HCV coinfection. Antivir Ther 2008; 13: 969-976
18. Harvey CE, Post JJ, Palladinetti P et al. Expression of the chemokine IP-10 (CXCL-10) by hepatocytes in chronic hepatitis C virus infection correlates with histological severity and lobular inflammation. J Leukoc Biol 2003; 74: 360-369
19. Diago M, Castellano G, Garcia-Samaniego J et al. Association of pretreatment serum interferon γ inducible protein 10 levels with sustained virological response to peginterferon plus ribavirin therapy in genotype 1 infected patients with hepatitis C. Gut 2006; 55: 374-379
20. Thompson AJ, Muir AJ, Sulkowski MS et al. Interleukin-28B Polymorphism improves viral kinetivs and is the strongest pretreatment predictor of sustained virologic response in genotype 1 hepatitis C virus. Gastroenterology 2010: 139: 120-129
21. Tanaka Y, Nishida N, Sugiyama M et al. Genome-wide association of IL28B with response to pegylated interferon-α and ribavirin therapy for chronic hepatitis C. Nat Genet 2009; 41: 1105-1109
22. Fabris C, Falleti E, Cussigh A et al. IL-28B rs12979860 C/T allele distribution in patients with liver cirrhosis: Role in the course of chronic viral hepatitis and the development of HCC. J Hepatol 2010; doi: 10-1016/j.hep.2010.07.019
23. Watashi K, Hijikata M, Hosaka M et al. Cyclosporine A suppresses replication of hepatitis C virus genome in cultured hepatocytes. Hepatology 2003; 38: 1282-1288.
24. Inoue K, Umehara T, Ruegg UT et al. Evaluation of a cyclophilin inhibitor in hepatitis C virus-infected chimeric mice in vivo. Hepatology 2007; 45: 921-928.
25. Nakagawa M, Sakamoto N, Enomoto N, et al. Specific inhibition of hepatitis C virus replication by cyclosporin A. Biochem Biophys Res Commun 2004; 313. 42-47
26. Pollard S: Calcineurin inhibition and disease recurrence in the hepatitis C virus-positive liver transplant recipient. Liver Int 2004: 24; 402-406
27. Chatterji U, Bobardt M, Selvarajah S et al. The isomerase active site of cyclophilin A is critical for hepatitis C virus replication. J Biol Chem 2009; 284: 16998-17005
28. Ma S, Boerner JE, TiongYip C et al. NIM811, a cyclophilin inhibitor, exhibits potent in vitro activity against hepatitis C virus alone or in combination with alpha interferon. Antimicrob Agents Chermother 2006; 50: 2976-2982
29. Paeshuyse JA, Kaul E, De Clercq B et al. The non-immunosuppressive cyclosporin DEBIO-025 is a potent inibitor of hepaitits C virus replication in vitro. Hepatology 2006; 43: 761-770
30. Hopkins S, Scorneaux B, Huang Z, et al. SCY-635, a novel non-immunosuppressive analog of cyclosporine that exhibits potent inhibition of hepatitis C virus RNA replication in vitro. Antimicrob Agents Chemother 2010: 54: 660-672.
31. Goto K, Watashi K, Murata T, et al. Evaluation of the anti-hepatitis C virus effects of cyclophilin inhibitors, cyclosporin A, and NIM811. Biochem Biophys Res Commun. 2006 12; 343: 879-884
32. Inoue K, Sekiyama K, Yamada M, et al. Combined interferon alpha2b and cyclosporin A in the treatment of chronic hepatitis C: controlled trial. J Gastroenterol 2003; 38: 567-572
33. Rayhill SC, Barbeito R, Katz D et al. A cyclosporine-based immunosuppressive regimen may be better than tacrolimus for long-term liver allograft survival in recipients transplanted for hepatitis C. Transplant Proc 2006; 38: 3625-3628
34. Selzner N, Renner EL, Selzner M et al. Antiviral treatment of recurrent hepatitis C after liver transplantation: Predictors of response and long-term outcome. Transplantation 2009; 88: 1214-1221
35. Nanmoku K, Imaizumi R, Tojimbara T, et al. Effects of immunosuppressant on the progression of hepatitis C in hepatitis C virus-positive renal transplantation and the usefulness of interferon therapy. Transplant Proc. 2008; 40: 2382-2385
36. Montori VM, Basu A, Erwein PJ et al. Posttransplantation diabetes. A systemic review of the literature. Diabetes Care 2002; 25: 583-592
37. Bodziak KA, Hricik DE. New-onset diabetes mellitus after solid organ transplantation. Transplant Int 2008; 22: 519-530
38. Ducloux D, Kazory A, Chalopin JM. Posttransplant diabetes mellitus and atherosclerotic events in renal transplant recipients: A prospective study. Transplantation 2005; 79: 438-443
39. Kasiske BL, Snyder JJ, Gilbertson D et al. Diabetes mellitus after kidney transplantation in the United States. Am J Transplant 2003; 3: 178-185
40. Hjelmesaeth J, Hartmann A, Leivestad T et al. The impact of early-diagnosed new-onset post-transplant diabetes mellitus on survival and major cardiac events. Kidney Int 2006; 69: 588-595
41. Woodward RS, Schnitzler MA, Baty J et al. Incidence and cost of new onset diabetes mellitus among U.S. wait-listed and transplanted renal allograft recipients. Am J Transplantation 2003; 3: 590-598
42. Rodrigo E, Fernandez-Fresnedo G, Valero R et al. New-onset diabetes after kidney transplantation: risk factors. J Am Soc Nephrol 2006; 17: 291-295
43. Kasiske BL, Snyder JJ; Gilbertson D et al. Diabetes mellitus after kidney transplantation in the United States. Am J Transplant 2003; 3: 178-185
44. Petta S, Cammà C, Scazzone C et al. Low vitamin d serum level is related to severe fibrosis and low responsiveness to interferon-based therapy in genotype 1 chronic hepatitis C. Hepatology 2010; 51: 1158-1167
45. Arteh J, Narra S, Nair S: Prevalence of Vitamin D deficiency in chronic liver disease. Dig Dis Sci 2010: 55: 2624-2628
46. Bitetto D, Fabris C, Fornasiere E et al. Vitamin D supplementation improves response to antiviral treatment for recurrent hepatitis C. Transplant Int 2011; 24: 43-50
47. Kayaniyil S, Vieth R, Retnakaran R et al. Association of vitamin D with insulin resistance and β-cell dysfunction in subjects at risk for type 2 diabetes. Diabetes Care 2010; 33: 1379-1381
48. Forouhi NG, Luan J, Cooper A, et al. Baseline serum 25-hydroxy vitamin D is predictive of future glycemic status and insulin resistance. Diabetes 2008; 57: 2619-2625
49. Foucher J, Chanteloup E, Vergniol J, et al. Diagnosis of cirrhosis by transient elastography (FibroScan): a prospective study. Gut 2005; 55: 403-408
50. Mari A, Pacini G, Murphy E, Ludvik B, Nolan JJ: A model-based method for assessing insulin sensitivity from the oral glucose tolerance test. Diabetes Care 2001; 24: 539-548
51. Kanauchi M, Yamano S, Kanauchi K, Saito Y: Homeostasis model assessment of insulin resistance, quantitative insulin sensitivity check index, and oral glucose insulin sensitivity index in noobese, nondiabetic subjects with high-normal blood pressure. J Clin Endocrinol Metab 2003; 88: 3444-3446
52. Kautzky-Willer A, Krssák M, Winzer C, Pacini G, Tura A, Farhan S, Wagner O, Brabant G, Horn R, Stingl H, Schneider B, Waldhäusl W, Roden M: Increased intramyocellular lipid concentration identifies impaired glucose metabolism in women with previous gestational diabetes. Diabetes 2003; 52: 244-251
53. Rask E, Olsson T, Söderberg S, Holst JjJ, Tura A, Pacini G, Ahrén B: Insulin secretion and incretin hormones after oral glucose in non-obese subjects with impaired glucose tolerance. Metabolism 2004; 53: 624-631
54. Ahrén B, Pacini G: Importance of quantifying insulin secretion in relation to insulin sensitivity to accurately assess beta cell function in clinical studies. European Journal of Endocrinology 2004; 150: 97-104
55. Thomaseth K, Kautzky‑Willer A, Ludvik B, Prager R, Pacini, G: Integrated mathematical model to assess beta‑cell activity during the oral glucose test. American Journal of Physiology 1996; 270: E522‑531
56. Tura A, Pacini G, Kautzky-Willer A, Ludvik B, Prager R, Thomaseth K: Basal and dynamic proinsulin-insulin relationship to assess beta-cell function during OGTT in metabolic disorders. American Journal of Physiology. Endocrinology and Metabolism 2003; 285: E155-162
57. Ahrén B, Pacini G: Importance of quantifying insulin secretion in relation to insulin sensitivity to accurately assess beta cell function in clinical studies. European Journal of Endocrinology 2004; 150: 97-104
58. Ahrén B, Pacini G: Impaired adaptation of first‑phase insulin secretion in postmenopausal women with glucose intolerance. American Journal of Physiology 1997; 273: E701‑707
59. Kahn SE, Prigeon RL, McCulloch DK, Boyko EJ, Bergman RN, Schwartz MW, Neifing JL, Ward WK, Beard JC, Palmer JP, Porte D Jr.: Quantification of the relationship between insulin sensitivity and beta‑cell function in human subjects. Evidence for a hyperbolic function. Diabetes 1993; 42: 1663‑1672
60. Stadler M, Anderwald C, Karer T, Tura A, Kästenbauer T, Auinger M, Bieglmayer C, Wagner O, Kronenberg F, Nowotny P, Pacini G, Prager R: (2006) Increased plasma amylin in type 1 diabetic patients after kidney and pancreas transplantation. A sign of impaired beta-cell function? Diabetes Care 2006; 29: 1031-1038
